# Identification and targeting of regulators of SARS-CoV-2–host interactions in the airway epithelium

**DOI:** 10.1126/sciadv.adu2079

**Published:** 2025-05-16

**Authors:** Brooke Dirvin, Heeju Noh, Lorenzo Tomassoni, Danting Cao, Yizhuo Zhou, Xiangyi Ke, Jun Qian, Sonia Jangra, Michael Schotsaert, Adolfo García-Sastre, Charles Karan, Andrea Califano, Wellington V. Cardoso

**Affiliations:** ^1^ Columbia Center for Human Development, Columbia University Irving Medical Center, New York, NY 10032, USA.; ^2^ Department of Genetics and Development, Columbia University Irving Medical Center, New York, NY 10032, USA.; ^3^ Department of Systems Biology, Columbia University Irving Medical Center, New York, NY 10032, USA.; ^4^ Herbert Irving Comprehensive Cancer Center, Columbia University Irving Medical Center, New York, NY 10032, USA.; ^5^ Institute for Systems Biology, Seattle, WA 98109, USA.; ^6^ DarwinHealth Inc., New York, NY 10018, USA.; ^7^ Department of Medicine, Pulmonary Allergy Critical Care, Columbia University Irving Medical Center, New York, NY 10032, USA.; ^8^ Department of Pharmacology, Columbia University Irving Medical Center, New York, NY 10032, USA.; ^9^ Department of Microbiology, Icahn School of Medicine at Mount Sinai, New York, NY 10029, USA.; ^10^ The Rockefeller University, New York, NY 10065, USA.; ^11^ Department of Systems Biology, J.P. Sulzberger Columbia Genome Center, Columbia University Irving Medical Center, New York, NY 10032, USA.; ^12^ Department of Biomedical Informatics, Vagelos College of Physicians and Surgeons, Columbia University, New York, NY 10032, USA.; ^13^ Department of Medicine, Vagelos College of Physicians and Surgeons, Columbia University, New York, NY 10032, USA.; ^14^ Chan Zuckerberg Biohub New York, New York, NY, USA.

## Abstract

The impact of SARS-CoV-2 in the lung has been extensively studied, yet the molecular regulators of host-cell programs hijacked by the virus in distinct human airway epithelial cell populations remain poorly understood. Some of the reasons include overreliance on transcriptomic profiling and use of nonprimary cell systems. Here we report a network-based analysis of single-cell transcriptomic profiles able to identify master regulator (MR) proteins controlling SARS-CoV-2–mediated reprogramming in pathophysiologically relevant human ciliated, secretory, and basal cells. This underscored chromatin remodeling, endosomal sorting, ubiquitin pathways, as well as proviral factors identified by CRISPR assays as components of the viral-host response in these cells. Large-scale drug perturbation screens revealed 11 candidate drugs able to invert the entire MR signature activated by SARS-CoV-2. Leveraging MR analysis and perturbational profiles of human primary cells represents an innovative approach to investigate pathogen-host interactions in multiple airway conditions for drug prioritization.

## Teaser

A network-based approach identifies master regulators of SARS-CoV-2 infection in airways and prioritizes drugs targeting.

## INTRODUCTION

The conducting airway epithelium is the first line of defense in the respiratory tract against pathogens and a prime site of severe acute respiratory syndrome coronavirus 2 (SARS-CoV-2) viral entry, also serving to maintain a high viral load during lung infection. Single-cell transcriptomics has been widely used to investigate viral-host interactions and to identify genes that may mediate interaction with viral proteins to support COVID-19 pathogenesis. Two main limitations have potentially affected the relevance of these studies. First, studies have initially explored nonphysiologic cell lines sensitized to SARS-CoV-2 infection by transgenic ACE2-TMPRSS2 expression (*1*). More critically, most studies have leveraged cell lines derived from nonlung sources or even cancer cell lines (*2*–*8*). Although they uncovered key mechanisms of viral-induced host responses associated with deleterious cellular effects, they had limited impact in revealing tissue- or cell type–specific disease mechanisms, especially as they relate to the highly heterogeneous lung- and airway-specific epithelium. To address these challenges, recent studies have used human primary cells grown in organotypic cultures or human induced pluripotent stem cell–derived lung organoids, thus enhancing the relevance of these screens (*9*).

Studies to elucidate the mechanisms by which SARS-CoV-2 hijacks the cellular function in the lung epithelium have relied largely on the analysis of single-cell transcriptomic profiles. Since gene expression may not represent a proxy of protein activity, these results are intrinsically biased toward the downstream effects of virus-mediated host-cell reprogramming rather than the key proteins—including transcription factors (TFs) and cofactors—that represent their upstream drivers. We and others have shown that these limitations can be effectively addressed by relying on network-based algorithms—such as Virtual Inference of Protein-activity by Enriched Regulon Analysis (VIPER) (*10*) and its single-cell extension metaVIPER (*11*)—that can accurately identify master regulator (MR) proteins of pathophysiologic gene expression signatures, via their transcriptional targets (*12*, *13*). This is especially relevant at the single-cell level where as many as 80 to 90% of genes may produce no reads, an effect known as gene dropout that greatly diminishes the ability to elucidate biological mechanisms from single-cell data. Specifically, by analyzing the differential expression of a protein transcriptional targets—as identified by the extensively validated Algorithm for the Reconstruction of Accurate Cellular Networks (ARACNe) (*14*)—VIPER assesses the contribution of every TF and co-TF to implementing a specific gene expression signature (henceforth protein activity). This allows for measuring the activity of proteins even when mRNA reads are minimally detected, making the algorithm especially well-suited for single-cell analyses. VIPER has been shown to outperform antibody-based expression analysis in single cells, because (i) protein abundance is a suboptimal proxy for protein activity and (ii) antibody availability and specificity is still limited. VIPER has been extensively applied to investigate pathogenetic mechanisms in cancer leading to successful clinical trials (*13*, *15*–*19*, *20*, *21*), as well as in noncancer-related fields, including immunology (*22*), diabetes (*23*), regenerative medicine (*22*, *24*), neurodegenerative disease (*25*–*27*), and stem cell biology (*22*, *28*, *29*).

Although we previously leveraged these network-based approaches to investigate SARS-CoV-2 (*30*), there were critical limitations. First, the analysis relied on cancer cell lines—a poor proxy for understanding the pathophysiologic responses of human lung epithelial cells; second, only the average effects of the virus on the multiple subpopulations that comprise the human lung epithelium were investigated rather than the subpopulation-specific responses. Third, the library of drugs used in these analyses was limited to oncology drugs with relatively high toxicity.

Here, we addressed these fundamental limitations by performing VIPER-based analyses of human airway cells, grown in organotypic cultures to identify the host-cell hijacking programs induced by SARS-CoV-2 in basal, ciliated, and secretory cells at the single-cell level and at different stages of infection. Integration of VIPER-based MR activity with recent CRISPR–knockout (KO) screens (*3*, *31*, *32*) revealed both pan-airway epithelial and airway cell type–specific MR modules controlling the regulatory programs hijacked by SARS-CoV-2. Analysis of cells directly infected with SARS-CoV-2 compared to bystander cells revealed non–interferon (IFN) signatures enriched in epigenetic regulators [histone deacetylases (HDACs)/DNMT], endosomal, ubiquitin, and other pathways with components of these signatures identified in all or in specific cell types. To identify small-molecule compounds capable of inverting these MR activities, we performed a large-scale perturbation screen in airway epithelial organotypic cultures using a library of Food and Drug Administration (FDA)–approved drugs not limited to oncology. This screen identified 11 drugs able to target SARS-CoV-2–mediated MR signatures across the three airways subpopulations. The network-based approach we used here (ViroTreat) (*30*) allowed targeting of the entire signature of MR proteins identified as mediators of the SARS-CoV-2 hijacking of the host cell programs rather than an individual regulator. This is highly advantageous in a heterogeneous primary cell system, such as the airway epithelium, as the individual proteins controlling these effects may be different in different subpopulations.

Overall, the framework described here can be broadly extended to investigate determinants of viral-host interaction and MR-mediated reprogramming by a variety of other pathogens affecting the human airway epithelium, as well as to prioritize clinically relevant drugs or agents as potential inhibitors. In particular, the drug perturbation assays—which represent the most expensive and time-consuming element of this study—provide a universal resource that can be leveraged to prioritize drugs targeting the host-cell MR signatures induced by virtually any other pathogen affecting lung epithelial cells, thus requiring only assessment of pathogen-specific gene expression signatures for MR analysis.

## RESULTS

### A network-based approach identifies common and cell type–specific pathways of the SARS-CoV-2–induced host response in the human airway epithelium

**Fig. 1.**
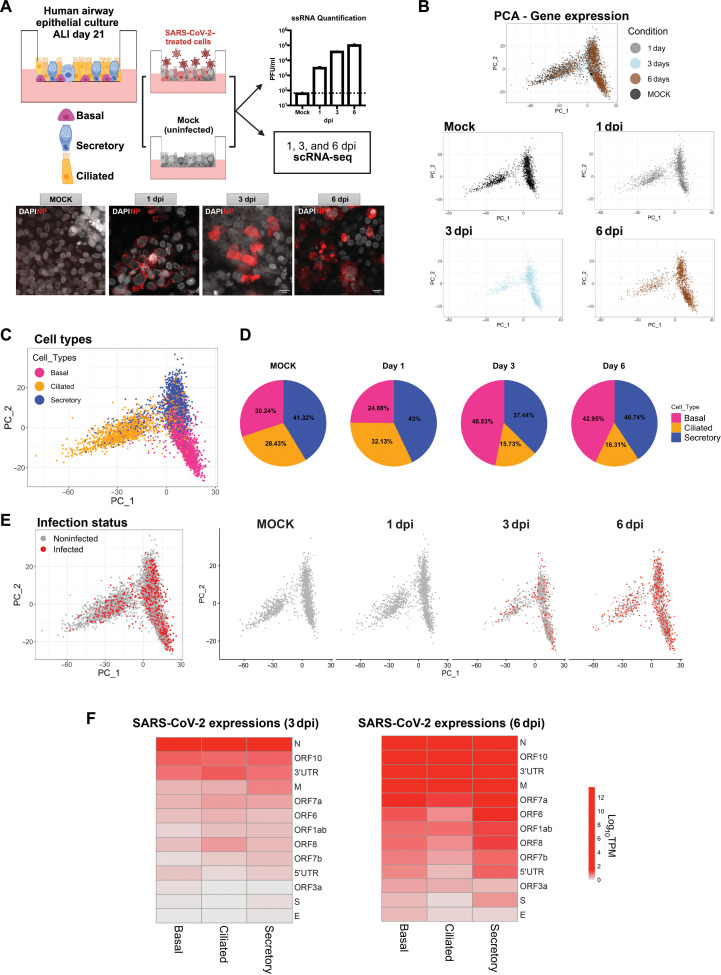
SARS-CoV-2 infects distinct adult human airway epithelial cell populations in ALI organotypic culture. (**A**) Diagram experimental design: Human airway epithelial cells from adult donors cultured in ALI for 21 days were infected with SARS-CoV-2 (MOI: 0.1) or untreated (mock) and processed for scRNA-seq at 1, 3, and 6 dpi. Graph: Quantification of single-stranded RNA [plaque-forming units (PFU)/ml, *n* = 3 cultures per time point]. Immunofluorescence and confocal imaging of cultures immunostained for the SARS-CoV-2 NP (red) protein and 4′,6-diamidino-2-phenylindole (DAPI; gray). Scale bars, 10 μm. (**B**) Principal components analysis (PCA) plots based on gene expression data of QC-filtered cells. Each dot represents a single cell for all four different conditions (top). Cells are also split into distinct subpanels: mock (black), 1 dpi (gray), 3 dpi (light blue), and 6 dpi (brown). (**C**) PCA plot based on gene expression data of QC-filtered cells. Cells are colored according to the different cell types: basal (pink), ciliated (orange), and secretory (blue). (**D**) Pie charts displaying the proportions of three cell types (basal, secretory, and ciliated cells) in the scRNA-seq data across time points (mock, 1, 3, and 6 dpi). (**E**) PCA plots based on gene expression data of infected (red) and noninfected (gray) cells from mock, 1 dpi, 3 dpi, and 6 dpi cultures. Infection status was determined by aligning raw data (FASTQ files) against the SARS-CoV-2 genome. Cells were considered infected if they contained at least one viral read mapped to the SARS-CoV-2 genome. (**F**) SARS-CoV-2 components: Heatmap showing the average gene expression values (log_10_TPM) of reads aligned to the viral genome for each cell type at 3dpi and 6dpi. 3′UTR, 3′ untranslated region; ssRNA, single-stranded RNA; N, nucleocapsid; M, membrane; S, spike; E, envelope.

To identify candidate regulators responsible of viral-induced hijacking of the host machinery and to investigate their corresponding biological functions, organotypic air-liquid interface (ALI) cultures from adult human extrapulmonary airways (lower trachea and main bronchi) were exposed to SARS-CoV-2 [USA/WA1 2020 strain, multiplicity of infection (MOI) 0.1, *n* = 3; Fig. 1A] or mock conditions (see Methods) after being cultured for 21 days. Control and infected cultures were then analyzed at 1, 3, and 6 days postinfection (dpi). Infection efficiency was confirmed by plaque assays and by the identification of nucleocapsid (NP) signals in immunofluorescence assays (Fig. 1A). Single-cell RNA sequencing (scRNA-seq) profiles were generated and analyzed to identify proteins controlling the programs hijacked by the virus in basal, ciliated, and secretory cells, as well as to assess their effect on cell function. Quality control (QC) filtering (table S1) revealed a minimal batch effect, with cells clustering according to the expected epithelial phenotypes, namely, basal, secretory, and ciliated cells, as assessed by analysis of established lineage markers and further validated via SingleR analysis (*33*) using previously reported single-cell profiles (Fig. 1, B and C, and fig. S1, A to C) (*34*).

Although the total number of epithelial cells was not significantly changed in the SARS-CoV-2–exposed and mock control cultures, the relative proportion of cell types differed over time in culture. The percentage of basal cells increased while that of ciliated cells decreased both at 3 dpi and 6 dpi, compared to controls (Fig. 1D; *P* ≤ 2.2 × 10^−16^, by chi-square test). Infection status was determined by alignment of quality-controlled cells against the SARS-CoV-2 genome, confirmed by at least one read per cell mapped to the viral genome. Consistent with the single-stranded RNA results, the viral genome alignment analysis showed a progressive increase in the proportion of infected cells versus noninfected cells at 3 and 6 days postexposure (Fig. 1E), with a progressive increase in the median number of viral reads in each cell type (fig. S2A). Our analysis also revealed *N* and *Orf10* among the most expressed viral genes in infected cells, across all cell types (Fig. 1F). In contrast to prior reports (*34*–*36*), no statistically significant differences in infection rates were detected across these three cell populations (table S2), a trend that was independent of the specific threshold of the mapped reads to the SARS-CoV-2 genome used for the identification of infected cells (fig. S2B).

**Fig. 2.**
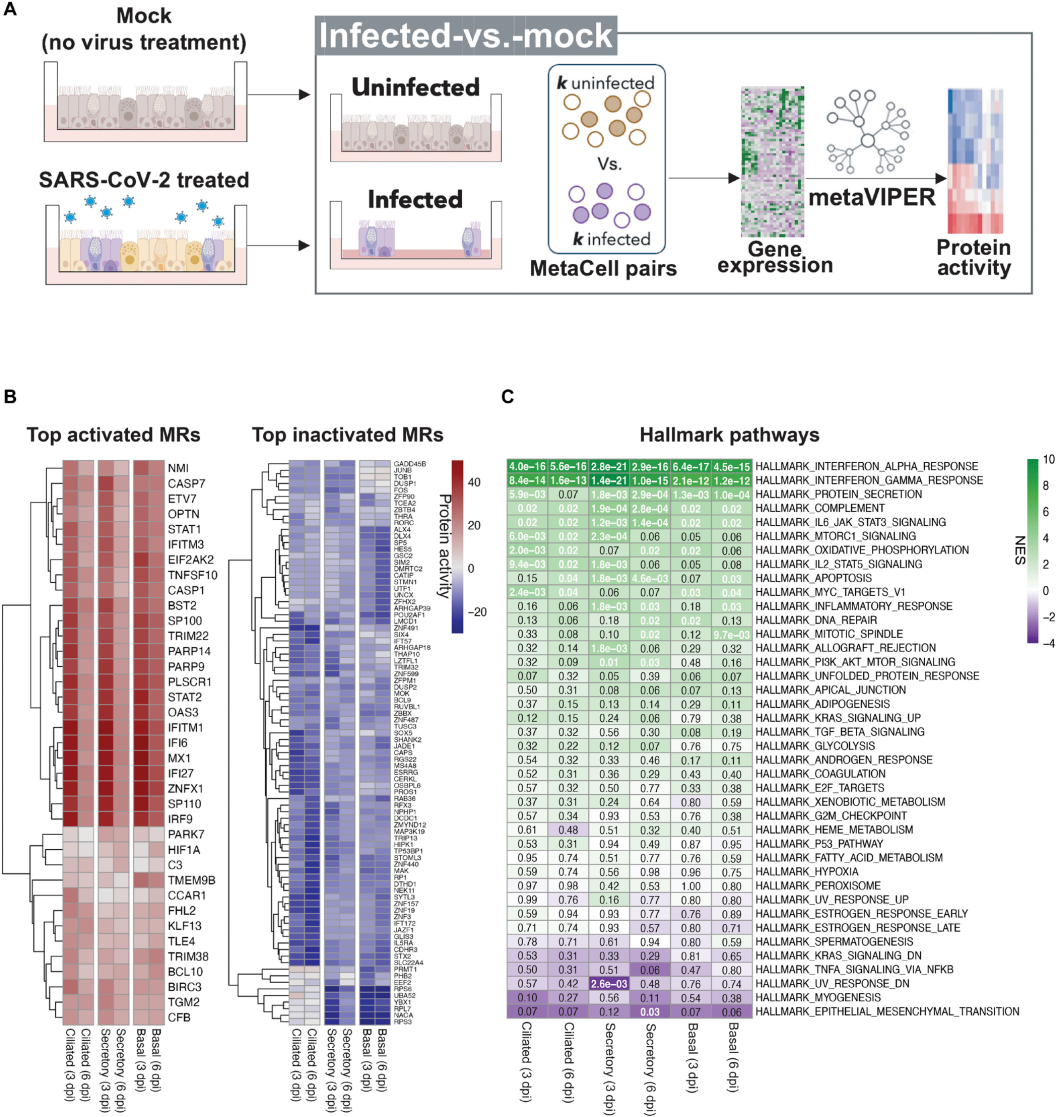
Protein activity analysis identifies regulators and targets of SARS-CoV-2 infection in human airway epithelial cells. (**A**) Diagram: Computational strategy for analysis of protein activity and MRs of SARS-CoV-2 infection from mock-versus-infected cultures. UIS, unspecific infection signature. Differential gene expression signatures of SARS-CoV-2–exposed (3 and 6dpi) versus mock (control) were generated for each cell type. Infected cells (*n* = *k*) versus mock cells (*n* = *k*) were randomly selected 100 times (i.e., repeated subsampling), and then their gene expression signature was converted computationally into protein activity using VIPER (see Methods). The protein activity signatures obtained throughout 100 subsamplings were then integrated using Stouffer’s method for each cell type. (**B**) Heatmap of the VIPER-inferred top differentially activated (left) and inactivated (right) proteins of SARS-CoV-2–infected versus mock cultures in each time point (3 and 6 dpi) and cell type (basal, secretory, and ciliated). Top activated and inactivated proteins were retrieved from each cell type signature and clustered, separately using the hierarchical clustering algorithm (average linkage method). Differential protein activity is shown as NES. Positive (brown) and negative (blue) values indicate protein activation and inactivation, respectively. (**C**) Heatmap depicting differential enrichment of biological hallmark pathways in SARS-CoV-2–infected versus mock protein signatures at 3 and 6 dpi. NES was estimated by aREA (see Methods). Enrichment of activated (purple) and inactivated (green) proteins is shown. The values inside the heatmap indicate Benjamini-Hochberg *P* values.

Next, we investigated the cell type–specific host response of ciliated, basal, and secretory cells by analyzing the differential activity of all regulatory proteins, as assessed by metaVIPER (*11*), the extension of VIPER to single-cell analyses. To generate host response signatures for metaVIPER analysis, we normalized the read counts using a metaCell approach, as described in Methods. As illustrated in Fig. 2A, for each metaCell, cell groups from the same condition were randomly selected and their read counts were merged. MetaVIPER (henceforth VIPER for simplicity) was then used to transform the differential gene expression signatures of individual SARS-CoV-2–infected metaCells at 3 and 6 dpi—versus metaCells from either mock controls or noninfected cells at the same time point—into differential protein activity profiles. For this purpose, we generated an airway-specific regulatory network by analyzing a publicly available repository of primary human airway epithelial gene expression profiles (*37*, *38*) with the ARACNe algorithm (*10*, *11*). The results of this analysis was a cell subpopulation–specific repertoire of proteins that were aberrantly activated or inactivated in response to SARS-CoV-2 infection in basal, ciliated, or secretory cells (Fig. 2B and fig. S2C).

Robustness of these observations was supported by comparative MR analysis of datasets from similar scRNA-seq studies of SARS-CoV-2–infected human ALI airway cultures. Among the several reports, we selected that by Ravindra *et al.* (*34*) because of the similarities with our study in multiple aspects, including the use of primary human bronchial epithelial cells, largely similar cell culture conditions, and protocols for processing and analyses. We generated host response signatures using the network-based computational framework described in our study. We focused our analysis on ALI at 3 dpi, a time point common to both studies, which showed SARS-CoV-2 infection and clear host responses across ciliated, secretory, and basal cells. MR signatures were derived from the differentially expressed genes in 3 dpi infected-versus-mock cultures and compared with our signatures [unspecific infection signature (UIS)] at 3 dpi. Enrichment analysis of the top 50 and bottom 50 MRs from both studies measured by a weighted enrichment analysis algorithm called analytic Rank-based Enrichment Analysis (aREA) (see Methods) (*10*) showed a high degree of conservation between these signatures when they were averaged across all cell types (*P* = 4.28 × 10^−19^) as shown in fig. S2D. A notably higher degree of conservation was observed when the analyses were performed in the basal (*P* = 6.92 × 10^−26^) and ciliated (*P* = 3.73 × 10^−42^) subpopulations, as summarized in fig. S2E. Secretory cells still produced a lower albeit still highly significant similarity (*P* = 3.48 × 10^−4^).

We reasoned that comparing infected cells to either mock or noninfected cells was critical to deconvoluting unspecific/indirect effects (cytokine secretion by infected cells affecting the noninfected bystanders) from the specific/direct effects (the reprogramming unique to the infected cells, where the unspecific effects are subtracted). Hence, we defined the signature derived from infected versus mock cells as the UIS, while the signature derived from infected versus noninfected bystander cells was termed the specific infection signature (SIS). As expected, hallmark (*39*) pathway analysis of the UIS signature revealed IFN-α/γ signaling, protein secretion, complement, and interleukin-6/Janus kinase/signal transducer and activator of transcription 3 (STAT3) signaling as the most significantly activated pathways in all cell types (Fig. 2C and fig. S3A). The 50 most differentially activated proteins included IFN-induced factors such as IFITM1—a known SARS-CoV-2 infection cofactor in the human lung (*40*)—IFI6, IFI27, MX1, interferon regulatory factor 9 (IRF9), STAT1, STAT2, as well as a number of others proteins (ZNFX1, PLSCR1, SP110, PARP14, TNFSF10, CASP1, and OAS3) that were prominently activated (*P* ≤ 0.001) in the host response signature (Fig. 2, B and C; fig. S3A; and table S3A) (*41*). When normalized enrichment scores (NES) were averaged over the three cell types at days 3 and 6, pathway enrichment analysis of the UIS signature—either based on differentially expressed genes or differentially active proteins—was highly concordant. Statistical significance of the concordance was assessed by Spearman correlation (ρ = 0.641, *P* = 8.2 × 10^−6^) and *F* statistic (*F* = 62.0, *P* = 1.7 × 10^−9^).

Results were also consistent with previous reports of significant innate immune response activation by SARS-CoV-2 in contexts as diverse as human airway–derived lung cancer cells (Calu-3), gastrointestinal organoids of different origins (*30*), and cultured human bronchial epithelial cell ALI cultures (fig. S3B) (*34*, *40*, *42*). Moving from pathway analysis to analysis of individual proteins, besides IFNs and related factors, the UIS also identified the zinc-finger protein ZNFX1 as a top activated MR in the three cell types analyzed, particularly at 3 dpi (Fig. 2B, left). Activation of ZNFX1 in immune cells isolated from bronchoalveolar lavage of patients with COVID-19 has been identified as an important component of the antiviral response (*41*). These previous studies indicated that ZNF proteins could directly recognize and bind to CpG sites in SARS-CoV-2 to induce IFN in infected cells in early stages of infection (*41*).

Thus, our findings of similar ZNFX1 activation in a system that consists solely of epithelial cells is noteworthy, suggesting that the ZNF-IFN association may occur in a broader cellular context. However, our MR analysis also revealed other ZNF family members among the top inactivated proteins in specific cell types. For example, ZNF491, ZNF157, and ZNF19 emerged from the UIS as significantly inactivated in ciliated cells compared to secretory or basal cells (*P* ≤ 0.01, by *t* test) (Fig. 2B, right). Whether the differential inactivation of ZNF family members selectively in specific cell types influences the susceptibility to viral infection remains to be investigated. There is also increasing evidence that viruses may hijack specific ribosomal proteins to achieve optimal viral translation (*43*). While broadly confirming these findings, our analysis also suggest that SARS-CoV-2 may selectively target the translational machinery in distinct cell types. For instance, we found specific ribosomal subunits (RPS6, RPS3, and RPL7) (*44*) and translation-related proteins [NACA (*45*), YBX1 (*46*), and PRMT1 (*47*)] to be significantly inactivated only in secretory and basal cells (*P* < 0.01 by *t* test) (Fig. 2B, right).

### Analysis of infected versus bystander signatures revealed cell type–specific SARS-CoV-2 host response MRs

Previous studies have shown that IFN signaling from virally infected cells, including SARS-CoV-2 infection, indirectly affects their immediate neighbor cells, thus contributing to a broad IFN signature activation across the entire airway epithelium (*34*). To account for potential indirect, paracrine effects, such as those induced by IFN signaling, we reanalyzed our data, now comparing infected (≥1 viral RNA reads) with noninfected bystander cells (with no viral RNA reads) in SARS-CoV-2–treated cultures, as captured by the SIS.Specifically, we reasoned that analysis of the SIS signature could identify additional, more specific host cell responses that otherwise could not be revealed by the UIS, in which infected and mock cells were analyzed without bystander cell contribution. The robustness of the SIS signatures was maintained despite the specific threshold of reads mapped to the viral genome used (fig. S3C) (see Methods).

**Fig. 3.**
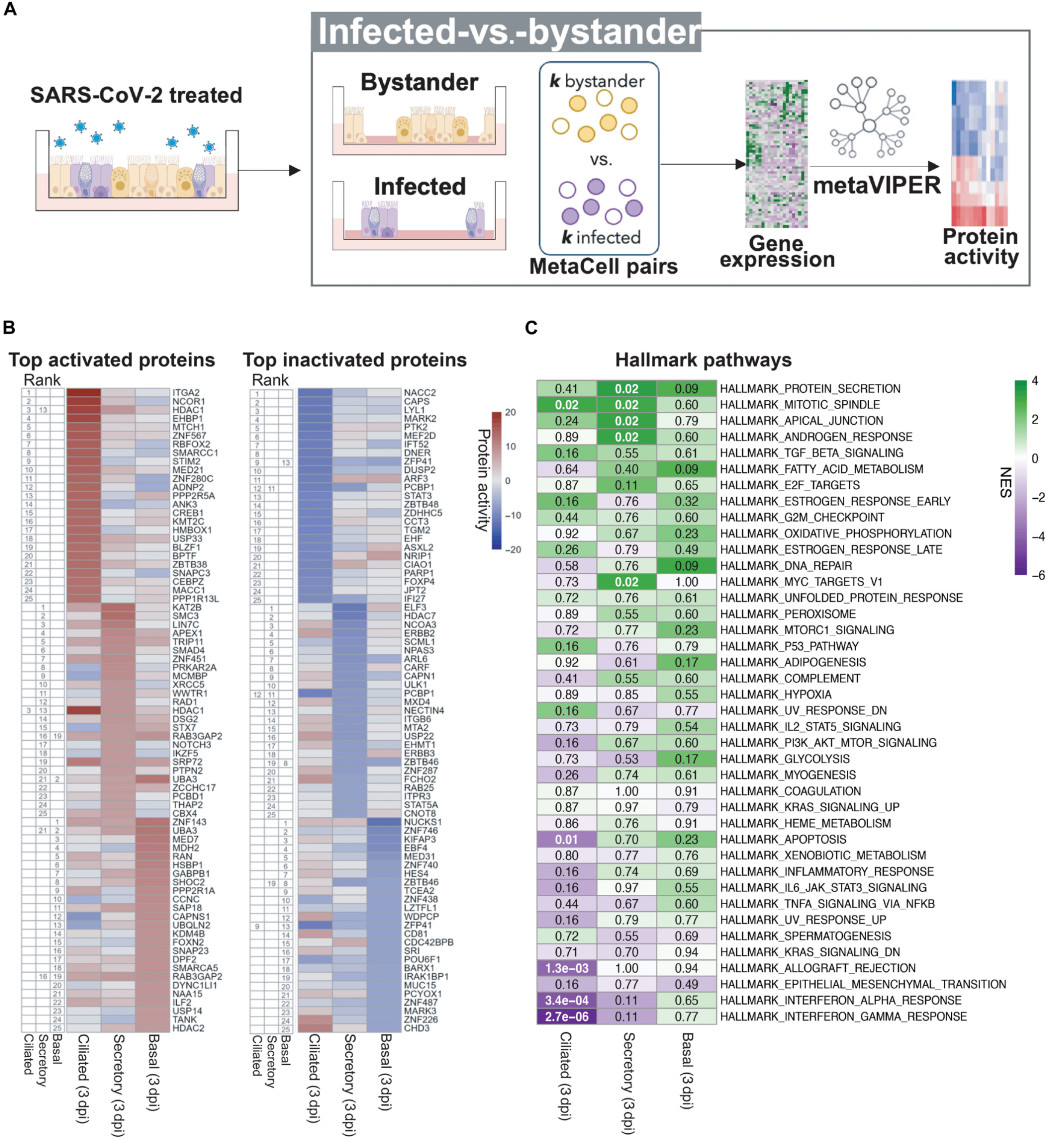
Protein activity analysis identifies the top MRs of the host response in distinct populations of human airway epithelial cells infected with SARS-CoV-2 compared to bystander cells (SIS). (**A**) Diagram computational strategy: Analysis of protein activity and MRs in infected versus bystander cells from SARS-CoV-2–treated cultures at 3 dpi. Infected (*n* = *k*) versus bystander (*n* = *k*) cells were randomly selected 100 times (as before), and then gene expression was converted into protein activity (VIPER, see Methods). The protein activity signatures obtained throughout 100 subsamplings were then integrated using Stouffer’s method for each cell type. (**B**) Heatmap of the VIPER-inferred top 25 differentially activated (left) and inactivated (right) proteins of SARS-CoV-2–infected versus bystander cultures at 3 dpi for each cell type analyzed. Ranking is shown numerically on the left of each heatmap. Differential protein activity is shown as NES. Positive (brown) and negative (blue) values indicate protein activation and inactivation, respectively. (**C**) Differential enrichment of biological hallmark pathways in SARS-CoV-2–infected versus bystander protein signatures at 3 dpi. NES was estimated by aREA (see Methods); activated (purple) and inactivated (green) proteins are shown. The values inside the heatmap indicate Benjamini-Hochberg *P* values.

For this, we focused on 3 dpi to minimize secondary effects (Fig. 3A). As expected, SIS analysis no longer highlighted “immune response” as differentially activated (Figs. 2, B and C, and 3, B and C). Instead, SIS identified MRs activated specifically in the infected cells, in some cases also revealing cell type specificity. For instance, hallmark features of SIS, such as protein secretion, apical junction, androgen response, and MYC target pathways, were significantly activated in secretory cells, while mitotic spindle was found activated mostly in ciliated and secretory cells (Fig. 3C). Hallmark estrogen response was found in basal and ciliated cells but not in secretory cells (Fig. 3C, fig. S3A, and table S3B). Viral infection is facilitated and propagated by heightened cell-to-cell transmission in apical junctions of the luminal mucociliary cells, with virion accumulation in the mucus layer, facilitating infection of neighboring cells (*48*–*50*). SARS-CoV-2 has also been proposed to hijack microtubule kinases crucial for organization of mitotic spindle to influence viral load and invasion. On the basis of this, anticancer drugs that block mitosis through disruption of microtubule organizing center have been tested in COVID-19 human clinical trials (*51*). Yet, since these effects appear to be largely cell population specific, these drugs may not be effective in a heterogeneous cellular context, thus underlining the relevance of the information provided by the SIS. The seemingly unrelated pathways described above collectively reflect the complex orchestration of events associated with COVID-19 pathogenesis in the airway epithelium, a system known for its significant cellular diversity.

At the individual protein level, analysis of the SIS signature identified ITGA2, NCOR1, and HDAC1 as the top three most activated host-cell proteins in ciliated cells following SARS-CoV-2 infection (Fig. 3B). ITGA2 encodes the α subunit of a transmembrane receptor integrin binding present in anchoring junctions to mediate cell-cell and cell-matrix interactions. A number of studies show that human adenovirus, herpesvirus, and other viral pathogens internalize into cells through a mechanism of integrin-mediated endocytosis (*52*, *53*). Notably, there is evidence of SARS-CoV-2 binding to Integrin and activation of endocytosis as an essential ACE2-independent component of the viral infection (*52*, *54*). ITGA2 has also been linked to p38 and p16-mediated senescence of SARS-CoV-2–infected cells (*55*).

The two additional MR proteins, representing key putative determinants of SARS-CoV-2 infection in ciliated cells, were NCOR1 and HDAC1. NCOR1 is part of a family of transcriptional corepressors shown to form complexes with both HDAC1 and HDAC2. Furthermore, HDAC family members identified in our screen included HDAC7 and HDAC2, among the most activated proteins in secretory cells and basal cells, respectively (Fig. 3B). KAT2B being the most aberrantly activated MR in infected secretory cells could further support the idea of a mechanism of SARS-CoV-2–induced chromatin remodeling. SMC3 (structural maintenance of chromosomes 3) emerged as the second most aberrantly activated MR in secretory cells. SMC3 is a key component of the cohesin complex, which plays a critical role in the regulation of chromosome structure and gene expression. Specific activation of these epigenetic regulatory pathways in infected secretory cells is intriguing and worth future studies.

Consistent with prior studies, VIPER analysis identified MRs representing key complementary subunits of the middle module of the mediator complex, including MED21 and MED7—aberrantly activated in ciliated and basal cells, respectively—and MED31—aberrantly inactivated in basal cells. This complex associates directly with RNA polymerase II to regulate its function (*56*). Biochemical structural data have shown that MED7 and MED21 form a hinge that enhances stable interactions between RNA polymerase II and the mediator complex, indicating that the activation of these proteins may be associated with increased transcriptional machinery activity (*56*). Together, the data suggested engagement of distinct SARS-CoV-2–mediated epigenetic reprogramming pathways in these populations of airway epithelial cells.

### Proviral host factors crucial for SARS-CoV-2 infection are selectively induced in different cell types

We then asked whether host factors previously reported to physically interact with SARS-CoV-2 proteins or shown to be critical for infection (proviral replication), based on CRISPR studies (*3*, *31*, *32*), were differentially enriched in MR proteins. Host factors co-opted by SARS-CoV-2 during infection have been previously investigated, using genome-scale CRISPR-KO screens, in multiple cell types including Vero.E6 (monkey) (*6*), Huh.7.5 (human hepatocarcinoma) (*4*), A549 (human lung adenocarcinoma) (*32*), and Calu-3 (immortalized human airway–like cell line derived from submucosal glands) (*3*, *31*). These screens helped identifying several genes, including ACE2 and HMGB1, whose inactivation increased the viability of infected cells compared to nontargeting single guide RNAs. From these studies, we selected the top 50 TFs and cofactors identified by CRISPR screens in A549 and Calu-3, based on their common origin from the lung/airway epithelium.

**Fig. 4.**
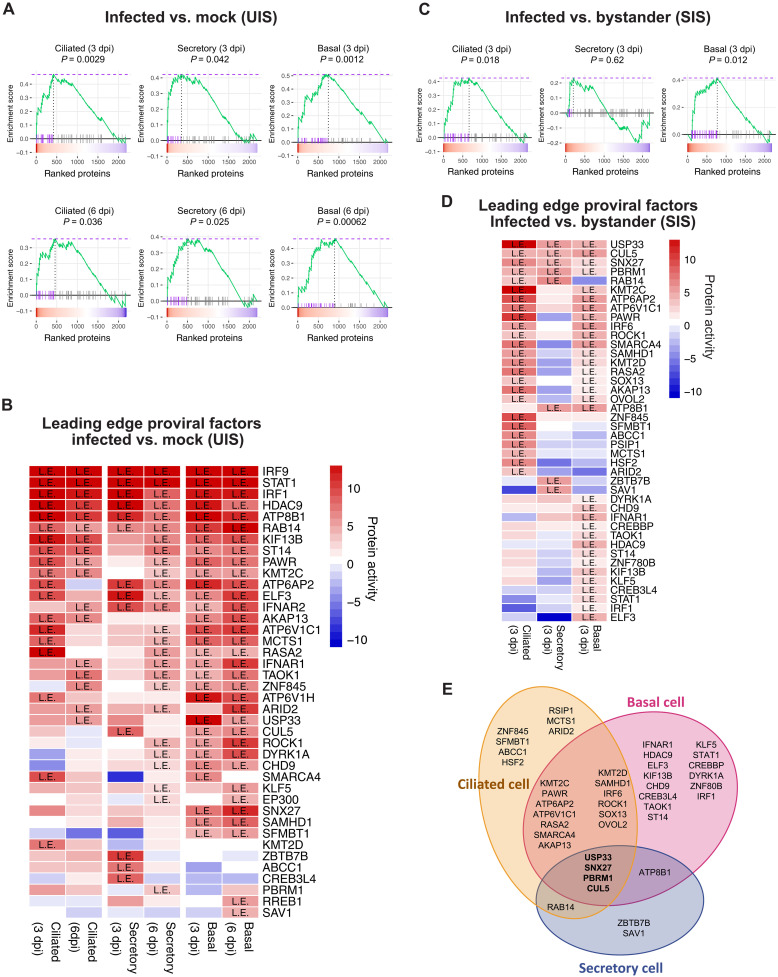
Proviral host factors crucial for SARS-CoV-2 infection are induced in different airway epithelial cell types. (**A**) Enrichment plots of proviral factors (*n* = 50) in the SARS-CoV-2–infected versus mock host response signature at 3 and 6dpi in each cell type. The color map on the bottom of each condition represents the host response signature (red; activated, blue; inactivated). Each bar represents the ranking enrichment of one proviral factor retrieved from previously identified gene sets from CRISPR-KO assays in human lung cancer cell lines (A549 and Calu-3). The green line represents the accumulative enrichment score. The red dotted line (top) shows the maximum enrichment score reached in GSEA. Hits on the left side of the vertical dotted line represent the subset of leading edge proviral factors among all 50 found in each condition. Enrichment *P* values were calculated by fast GSEA (fGSEA) (*86*). (**B**) Heatmap of protein activity (red: positive; blue: negative) of proviral factors found in the leading edge (L.E.) of SARS-CoV-2–infected versus mock GSEA plots from each condition. Rows were sorted in decreasing order of leading edge frequency across signatures. (**C**) Enrichment plots of proviral factors (*n* = 50) in the SARS-CoV-2–infected versus bystander host response signature at 3 dpi in each cell type. *P* values were calculated by fGSEA, and leading edge subsets were represented as in (A). (**D**) Heatmap of protein activity of proviral factors at the leading edge of SARS-CoV-2–infected versus bystander GSEA plots in each cell type at 3 dpi. Rows were sorted in decreasing order of leading edge frequency across signatures. (**E**) Venn diagrams summarizing the overlap of leading edge host factors identified in (A) among the three cell types at 3 dpi.

UIS analysis recapitulated several of these proviral factors among the 50 most differentially active proteins (i.e., candidate MRs of host-cell hijacking, hereafter MRs for simplicity) across all cell types, at both 3 and 6 dpi (Fig. 4A). Leading edge analysis revealed IRF1, IRF9, and STAT1 as the topmost conserved factors across all three cell populations (Fig. 4B). The identification of IFN regulatory factors and core transcriptional regulators of the inflammatory response as leading edge proteins—i.e., proteins identified among the most significant MRs but also positively modulating virulence, as supported by previously published CRISPR screens (*3*, *31*, *32*)—was intriguing as it suggested a dual role for these proteins as both proviral and antiviral factors during SARS-Cov-2 infection. Additional top leading edge proviral factors identified by UIS signature analysis included MRs of distinct functional classes, including several previously implicated in viral internalization. Among these, four adenosine triphosphatase (ATPase) and accessory proteins family members (ATB8B, ATP6AP2, ATP6V1C1, and ATP6V1H) were among the most aberrantly activated. Studies using bafilomycin to inhibit vacuolar ATPase (*57*) suggest that these ATPases act as proviral factors by facilitating SARS-CoV-2 entry through the endosomal route. This is further supported by our findings of the kinesin KIF13B and the small guanosine triphosphatase (GTPase) RAB14, known regulators of intracellular trafficking, vesicle formation, and endosomal recycling (*58*, *59*), as key leading edge MRs (Fig. 4B).

Next, we examined which of the top 50 proviral factors were also nominated as MRs by the SIS analysis. USP33, CUL5, sorting nexin 27 protein (SNX27), and PBRM1 emerged as the only leading edge proviral factors identified as activated MRs in all the three cell types. (Fig. 4, C to E). Two of these factors have been functionally implicated in the regulation of ubiquitination in viral infection. USP33 is a deubiquitinase enzyme reported to modulate the host inflammatory response and antiviral activity through regulation of the turnover of IRF9 (*60*). CUL5, a component of the Cullin-5-RING E3 ubiquitin-protein ligase complex, has been shown to act as a critical antiviral host factor promoting ubiquitin-dependent degradation of the SARS-CoV-2 ORF9b protein (*61*). The SNX27 controls cargo recycling from endosomes to the cell surface inhibit viral lysosome/late endosome entry by regulating ACE2 abundance. SNX27 can be hijacked by SARS-CoV-2 to facilitate viral entry (*62*). PBRM1 is a chromatin modulator of the SWI/SNF chromatin remodeling complex, which includes *SMARCA4*, *SMARCB1*, *SMARCC1*, *ARID1A*, *DPF2*, and *SMARCE1*, also implicated in *ACE2* expression regulation and, thus, in host cell susceptibility to viral infection (*63*).

These data reinforce the hypothesis that epigenetic reprogramming represents a key regulatory process leveraged by SARS-CoV-2 for hijacking host-cell programs in the airway epithelium. The analysis also helped identifying proviral factors that are either preferentially activated in a specific SARS-CoV-2–infected subpopulation or those shared across different subpopulations. Among the 50 leading edge proviral factors present in the SIS signature, most were found activated in ciliated, basal cells, or in both populations but not in secretory cells (Fig. 4, D and E). Comparative analysis of the SIS and UIS MR signatures confirmed a lower number of proviral factors differentially activated in secretory cells (fig. S4). However, it is important to note that SARS-CoV-2 induces a robust MR activation selectively in secretory cells, as demonstrated by the SIS analysis of this subpopulation (Fig. 3B).

The fact that our analysis identified key proteins known to modulate SARS-CoV-2 infection suggests that top cell population–specific MRs, as well as those conserved across multiple cell populations, that were not previously characterized may represent key regulators of undescribed processes of host-cell hijacking by the virus, for future low-throughput validation assays. Moreover, the diversity of factors revealed by these analyses further reinforced the idea that the signature elicited in the airway epithelium by SARS-CoV-2 represents a complex combination of host defense and virus-hijacked signals.

### A large-scale functional drug screen identifies candidate drugs that effectively invert the SARS-CoV-2–induced MR signatures in the human airway epithelium

To identify drugs capable of inverting the MR activity signature induced by SARS-CoV-2 infection, we performed a large-scale drug perturbation screen in these organotypic airway epithelial cell cultures, thus supporting prediction of candidate MR inverter drugs using the ViroTarget and ViroTreat (*30*) algorithms. These represent the direct extension to the viral infection context of OncoTarget (*18*, *20*) and OncoTreat (*18*, *19*, *64*), two CLIA-compliant (https://cms.gov/medicare/quality/clinical-laboratory-improvement-amendments) algorithms extensively validated in both preclinical and clinical oncology contexts. Drugs predicted as MR activity inverters by OncoTreat, from both bulk (*18*, *64*) and single-cell (*65*) profile data, have been validated by rigorous in vitro and in vivo assays. Consistent with this premise, we proposed that pharmacologic targeting of either individual MRs (ViroTarget) or of the entire MR protein module that regulates the virus-induced host-cell response (ViroTreat) could mitigate viral replication and infection comorbidity. Briefly, ViroTarget analyzes the list of MRs to assess whether any of them may represent a high-affinity binding target of a clinically relevant drug, among the 1738 in DrugBank (*66*). In contrast, ViroTreat assesses inversion of the virus-induced MR activity signature by assessing the enrichment of the 25 most activated and 25 most inactivated viral infection MRs in proteins differentially inhibited and activated in drug versus vehicle control–treated cells, respectively. For this analysis, we used the 3 dpi SIS signatures, representing a stage in which hijacking of host cell programs by the virus was likely to be fully established.

ViroTarget identified 32 MRs (*P* ≤ 0.05, Benjamini-Hochberg corrected) aberrantly activated in at least one of the infected cell types (ciliated, basal, or secretory cells)—as high-affinity targets of 87 small-molecule inhibitors in DrugBank (fig. S5). Druggable proteins associated with epigenetic control of gene expression, such as the HDAC family members HDAC1, HDAC2, HDAC3, and HDAC9, were identified as SIS MRs in distinct cell types. Consistently, HDAC inhibitors, such as romidepsin identified by ViroTarget, were already proposed as antiviral drug candidates (*30*, *67*). Additional MRs representing chromatin remodeling enzymes include EZH2, DNMT1, and CHD1; the latter is a chromatin organization modifier implicated in the recruitment of influenza virus polymerase to promote viral multiplication (*68*). These findings underscore the relevance and need for further investigation of epigenetic mechanisms hijacked by SARS-CoV-2 to induce host-cell reprogramming. ViroTarget also identified two of three RXR retinoid receptors as candidate druggable MRs, as also supported by independent evidence from the functional analysis of SARS-CoV-2–infected human induced pluripotent stem cell–derived organoids (*9*). Another notable druggable MR identified by ViroTarget was KRAS. This protooncogene found frequently mutated in human cancers has been implicated in viral stress responses mediated by GRP78, a chaperone induced by SARS-CoV-2 infection (*69*, *70*).

**Fig. 5.**
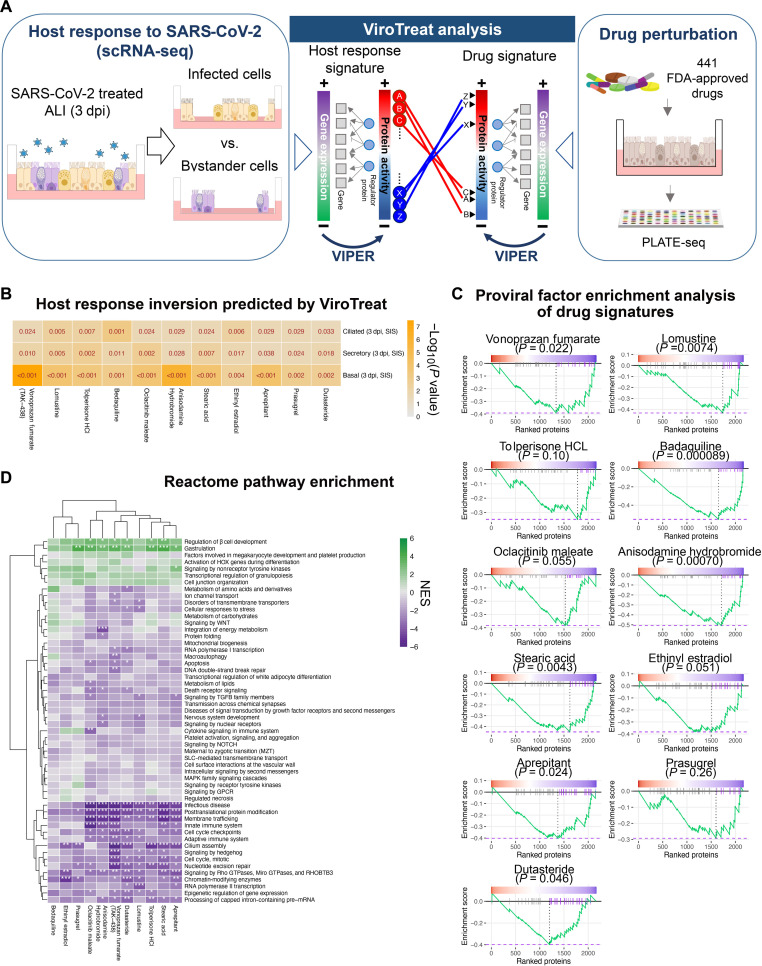
Pharmacologically targeting regulators of SARS-CoV-2–host interactions in the airway epithelium. (**A**) Schematic representation of the ViroTreat workflow approach. A high-throughput drug perturbation screen was performed by treating ALI day 21 human airway epithelial organotypic cultures with 441 FDA-approved drugs. Gene expression signatures were determined by PLATE-seq, converted to protein activity using VIPER and compared to the signatures previously obtained from SARS-CoV-2–infected versus bystander cells. The ViroTreat algorithm was used in these paralleled signatures to identify candidate drugs predicted to invert the activated (yellow) or deactivated (purple) host response signature of infected cells. (**B**) Heatmap of statistical significance (−log_10_ *P* value) for the top 11 drugs predicted by ViroTreat as strong inverters of the SARS-CoV-2 host response signatures from infected versus bystander cells at 3 dpi. The smaller *P* value corresponds to a stronger inverter of the host response. (**C**) Gene set enrichment plots of drug signatures. The gene set consisted of proviral factors which were included in the leading edge subsets for host responses at least for one cell type (*n* = 42). The negative enrichment score curve (green) indicates that the proviral set was negatively enriched in the drug signatures able to invert the host response signatures. (**D**) Heatmap representing the reactome pathway enrichments (NES) in the signature of the top 11 drugs. The positive (green) and negative (purple) NES indicate the activated and inactivated pathways. Rows and columns of the heatmap were sorted on the basis of a complete linkage hierarchical clustering method. *P < 0.05, **P < 0.01, and ***P < 0.001. MAPK, mitogen-activated protein kinase; GPCR, G protein–coupled receptor; WNT, wingless-related integration site; TGFB, transforming growth factor beta; SLC, small latent complex.

ViroTreat analysis requires a large-scale compendium of RNA-seq profiles representing the response of cells to treatment with a large library of clinically relevant compounds. For this purpose, we used the pooled library amplification for transcriptome expression sequencing (PLATE-seq) technology and VIPER, developed by our labs (*10*, *11*, *71*), to generate protein activity from the RNA-seq profiles of airway epithelial cultures treated with 441 FDA-approved drugs. We selected drugs with well-characterized bioactivity, safety, and bioavailability properties, as determined by preclinical and clinical studies. VIPER analysis of RNA-seq profiles of drug versus vehicle control–treated cells helps characterize the proteome-wide mechanism of action (MoA) of each drug, which can be used to assess the drug’s ability to invert the activity of the MR signatures identified from VIPER analysis of the SIS signature of each individual subpopulation (Fig. 5A) (see Methods). Here, we define MoA as the repertoire of proteins that are differentially activated or inhibited by a drug in a tissue of interest, including high-affinity binding targets, secondary lower-affinity targets, and context-specific indirect targets. Together, these proteins define the drug’s pharmacologic activity (*18*).

Drugs were added to differentiated ALI day 21 airway organotypic cultures at the their *C*_max_ concentration, and RNA-seq profiles were generated at 24 hours posttreatment, using the fully automated PLATE-seq technology (*71*) (see Methods) and analyzed by VIPER. Of 441 drugs, ViroTreat identified 11 as statistically significant MR inverters across all three airway epithelial cell types (Fig. 5B; figs. S6, A and B; and table S4). We further investigated the enrichment of MR in the leading edge of prior CRISPR screens, as described in the previous section (Fig. 4, D and E). ViroTreat analysis showed leading edge MR activity inversion in nearly all cell types and in 9 of 11 drugs (Fig. 5C and fig. S7A). Notably, bedaquiline—an established inhibitor of adenosine triphosphate (ATP) synthase in drug-resistant pulmonary tuberculosis (*72*)—was identified as a candidate inverter of USP33 and CUL5 activity.

Pathway enrichment analysis using gene sets from reactome pathway (*73*) showed that 8 of the 11 drugs identified by ViroTreat also induced statistically significant negative NES for pathways associated with virus-induced processes, including membrane trafficking, infectious diseases, posttranslational protein modification, and Rho GTPase signaling (Fig. 5D). Among the MRs whose activity was inverted by these drugs, we found SMC3 and MED7 (see the previous sections), as well as CREB1, USP33, ZFK451, SMARCA5, and others proteins reported in SARS-CoV-2–host interactions and pathogenesis databases (fig. S7B) (*58*). ViroTreat also identified USP33, PAWR, ATP6AP2, CUL5, ROCK1, and RAB14 as proviral MRs inactivated by 11 of the 411 drugs screened. While most of the factors targeted were enriched in ciliated cells, two of them (CUL5 and USP33) were present in all cell types (fig. S7A). Thus, the effect of ViroTreat-inferred MR inverter drugs extends to proteins controlling pathways associated with viral replication. Hence, these drugs should not only mitigate the hijacking of host-cell programs but also directly restrict viral replication.

## DISCUSSION

Here, we presented a multipronged approach for elucidating candidate MR proteins that are determinants of SARS-CoV-2–mediated hijacking of host cell programs, as well as to identifying the drugs that can invert their activity. This highly generalizable approach integrates single-cell network-based analyses and large-scale drug perturbation assays in physiologically relevant ALI organotypic cultures of human airway epithelial cells representing the distinct subpopulations that comprise the airway epithelium. The study supports key mechanisms identified in previous studies and nominates critical cell population–specific MR proteins controlling host-cell hijacking by the virus to improve replication and viral life cycle (ViroCheckPoint) as well as the drugs that can inhibit their activity for follow-up validation. As extensively validated in many studies, since protein activity is computed on the basis of the coherent differential expression of a large number of transcriptional targets of regulatory proteins (regulons), VIPER-nominated MRs are expected to represent a more robust measure of the host-cell response to viral infection, compared to gene expression. The latter can be noisy and highly sparse, especially in scRNA-seq profiles, due to gene dropout effects caused by low sequencing depth. Critically, the combination of VIPER-based MR nomination and large-scale, PLATE-seq–based drug perturbations profiling in human airway organotypic cultures allowed identification of FDA-approved drugs representing optimal MR protein activity inverters in SARS-CoV-2–infected cells.

The cell population–specific host signatures identified by this analysis characterizes a complex interplay between innate antiviral defense responses and proviral host-cell components hijacked by the virus. Distinguishing these two facets remains a substantial challenge due to their ambiguous dual functions (antiviral or proviral activities). Moreover, it is noteworthy that in the reactome pathway enrichment analysis, we found “innate immune system” responses significantly deactivated in the infected cells compared to bystanders. In contrast, the “cytokine signaling in immune system” pathway, which includes IFNs and related factors, was not significantly affected, either positively or negatively, by some of the drugs identified by ViroTreat analysis (Fig. 5D). Top candidate MR activity inverter drugs—such as vonoprazan fumarate, aprepitant, and anisodamine hydrobromide—induced inactivation of innate immune system but not “cytokine signaling” pathways, suggesting potential involvement of alternative mechanisms other than IFN-mediated. Our results suggest that the approach we report here using SARS-CoV-2–infected organotypic cultures reveals pathologic immune responses of the epithelium to viral infection likely to reflect those associated with barrier function processes. However, we expect these to be fundamentally distinct from the immune responses reported in patients with active severe late-stage disease.

Our analysis highlighted epigenetic modulators as potentially relevant determinants of the SARS-CoV-2–host responses. HDAC inhibitors have been reported to repress ACE2 expression and other genes responsible for viral entry. We identified the transcriptional corepressor NCOR1 and HDAC1 enriched in SARS-CoV-2–infected ciliated cells. NCOR-HDAC complexes are known to form complexes in vitro and in vivo to repress gene expression. Yet, the specific mechanisms by which NCOR-HDAC in ciliated cells promotes viral entry are not fully understood (*74*, *75*). HDAC2 has been shown to interact with NSP5, the main SARS-CoV-2 protease to inhibit HDAC2-mediated IFN responses (*2*). Our finding of the lysine acetyl transferase KAT2B as the most aberrantly activated MR in infected secretory cells could further support the idea of a mechanism of SARS-CoV-2 repression of IFN-related gene expression via chromatin remodeling to increased virulence. Last, SMC3 is reported as a nonhistone substrate of HDAC in cancer and has been shown to be involved in the restructuring of the chromatin architecture in SARS-CoV-2 infection (*76*, *77*).

ViroTreat identified 11 drugs as potentially able to rescue the normal state of regulatory host cell programs hijacked by SARS-CoV-2 infection in the airway epithelium. Notably, four of these drugs (bedaquiline, tolperisone HCl, prasugrel, and anisodamine), selected by our unbiased analysis, were also independently identified by protein-docking analyses as high-affinity binders of Mpro, the main protease of SARS-CoV-2 (*78*). Since our drug perturbation studies were not performed in the presence of the virus, this suggests that, in addition to interfering with Mpro—thus playing a crucial direct role in modulating viral replication—these drugs may facilitate additional processes to attenuate COVID-19 pathological burden, i.e., by both rescuing physiologic regulatory programs in infected cells and by attenuating the proteolytic activity of SARS-CoV-2. Notably, 2 of the 11 drugs identified by our approach (aprepitant and dutasteride) have been independently selected for COVID-19 clinical trials. Although none of these two have been found to be efficacious in these trials, their identification by our approach confirms the predictive efficacy of the ViroTreat algorithm as a screening tool for the identification of drug candidates for therapeutic intervention. However, it is important to emphasize that the methodology we report is a resource to interrogate questions about pathogen-host interactions and barrier function and serves as a first step to screen for candidate drugs that can invert viral-induced signatures in the airway epithelium. Multiple factors need to be considered for more precise drug selection and effective use in clinical settings, including investigation of dosing, chemical modification, formulation and others.

A key advantage of our approach is that, rather than targeting viral components that are under substantial mutational stress and may adapt rapidly to avoid neutralizing antibodies or viral-protein inhibitors, we target the host-cell system to reduce virus ability to replicate and counteract host mediators of the adverse epithelial response to virus. This is an important aspect, considering the rapid evolution of SARS-CoV-2 and its newer Omicron variants, which have been shown to have increased transmissibility, as well as accelerated and unique transcriptional responses as compared to the USA/WA1 2020 strain used in our study.

Together, our approach using relevant primary airway culture system and network-based computational approaches to identify SARS-CoV-2–host interactions and pharmacological targets is likely to have a broader application as a platform to study the effect of various viruses in the respiratory epithelium. Moreover, the gene expression profiles generated by drug perturbation of organotypic cultures of primary human airway epithelial cells represent a novel resource to identify candidate MR inverter drugs for infection by newer SARS-CoV-2 subvariants or any airway-specific pathogen, conditional only to the availability of infected versus noninfected cell signatures.

## METHODS

### Human airway epithelial organotypic air-liquid-interface (ALI) cultures

Primary human airway epithelial progenitors (basal cells) were isolated from deidentified adult human lung transplant organ donors in compliance with guidelines established by the International Institute for the Advancement of Medicine. Airway progenitors from regionally distinct sites (upper, lower trachea, and extrapulmonary main bronchus) were collected from different donors and differentiated in ALI organotypic cultures. Samples from lower trachea were used for these assays. Basal cells were cultured under submerged conditions in Pneumacult-Ex Plus expansion media (catalog no. 05040). After expansion, human airway epithelial cells were dissociated with TrypLE (ThermoFisher, #12604013) and plated (5 × 10^4^ cells) on collagen I–coated Transwells (0.4-μm pore; Corning, #354236) and subsequently replated in 24-well Transwell plates cultured in PneumaCult Ex-Plus media under submerged conditions until confluency (day 7). Differentiation was induced by removing the medium from the top chamber and adding Pneumacult-ALI media (catalog no. 05001) to the bottom chamber. Medium was changed every other day until ALI day 21, when cultures were fully differentiated, as previously described (*34*, *35*).

### SARS- CoV-2 infection of airway epithelial organotypic cultures

ALI day 21 airway epithelial organotypic cultures were inoculated with SARS-CoV-2 (USA/WA1-2020, NR-52281) on the apical surface at a previously tested MOI of 0.1. After 24 hours, the virus-containing medium in the apical chamber was removed, and cells were washed twice with phosphate-buffered saline (PBS) and cultured for 1, 3, and 6 dpi. Mock infection conditions at days 1, 3, and 6 were performed in parallel using the same reagents without virus. Plaque assays were performed for quantitation of viral infection as previously reported in (*79*). Briefly, the supernatant from the apical chamber of the Transwell culture plates was collected at the day of cell harvest and diluted to a concentration gradient at the power of 10 in 1× PBS containing 1% bovine serum. Samples were overlaid on preseeded VeroE6 cell monolayers in 2% Oxoid agarose mixed with 2× minimum essential medium 0.3% fetal bovine serum (FBS) and incubated for 72 hours at 37°C. After fixation, plaques were visualized by immunostaining with SARS-CoV-2 NP antibody (1C7). All SARS-CoV-2 infection experiments were conducted in a BSL-3 facility (Icahn School of Medicine at Mount Sinai, NY).

### Immunofluorescence

Transwell membrane inserts from human ALI organotypic cultures were fixed in 4% paraformaldehyde in PBS at room temperature for 1 hour (day 0) or overnight (day 28) and processed as reported in (*80*). The inserts were cut into six to eight pieces, blocked with 1% bovine serum albumin (Sigma-Aldrich, #A3294) and 0.5% Triton X-100 (Sigma-Aldrich, #9002-93-1) for 1 hour at room temperature. For immunofluorescence, samples were incubated with anti–SARS-CoV-2 nucleoprotein primary antibody (CTAD Mount Sinai, #NP-1C7) in 1% bovine serum albumin (Sigma-Aldrich) and 0.5% Triton X-100 at 4°C overnight and then washed with PBS and incubated with Alexa Fluor–conjugated secondary antibodies (1:300) and NucBlue Live Cell ReadyProbes Reagent (4′,6-diamidino-2-phenylindole) (Life Technology) for 1 hour. After washing, samples were mounted with ProLong Gold antifade reagent (Life Technology).

### Sample preparation for scRNA-seq

The apical chamber of Transwell plates containing either infected or mock-infected cells was washed two times with PBS at 37°C for 5 min. PBS was then removed, and 500 μl of accutase containing 5 mM EDTA was added into the apical and basolateral sides of the Transwells. The cells were gently pipetted up and down, collected into a 15-ml conical tube, and centrifuged at 1500 rpm for 3 min at 4°C. The medium was aspirated, and cells were resuspended in 500 μl of Dulbecco’s modified Eagle’s medium + 10% FBS and filtered through a 0.4-μl cell strainer into a 1.5-ml tube. Cells were then counted and diluted to 1 million cells/ml and placed on ice and sent for scRNA-seq (10X Genomics).

### scRNA-seq preprocessing and annotation of cell types and infection status

scRNA-seq FASTQ files were aligned to the human reference genome (GRCh38-2020-A) using 10x Genomics CellRanger software (v 5.0.1) to generate raw count matrices for all the four different experimental conditions, mock, 1 dpi, 3 dpi, and 6 dpi. Before proceeding with downstream analyses, stringent QC filtering metrics were applied to the data to remove low-quality cells. Specifically, cells with less than 30% content of mitochondrial genes and more than 1000 unique molecular identifiers (UMIs) were retained. After QC filtering, the average number of unique molecular reads per cell (UMIs per cell) was 2,723.742, and the average number of detected genes per cell was 1,053.218. Cells were labeled as either a basal, secretory, or ciliated cell according to well-established marker genes for each epithelial cell subtype. As independent confirmation of the cell types inferred using GSEA, we have also trained SingleR to annotate our single-cell data from mock condition using reference signatures published in (*34*). To quantify the number of infected cells in each experimental condition, reads in the FASTQ files were remapped to the SARS-CoV-2 reference genome (NC_045512.2) from GeneBank (*81*). For this, we used Kallisto Bustools (*82*) to estimate gene expression at the single-cell level. Infected cells were initially identified by detecting at least one read in any SARS-CoV-2 gene. Also, we analyzed the proportion of infected cells across the three cell types as viral read thresholds increased. At each threshold, a consistent proportion of infected cells was observed.

### Airway network inference from microarrays and scRNA-seq data

We generated gene regulatory networks specific for human airways using an ARACNe algorithm with adaptive partitioning (ARACNe-AP) (*83*). Briefly, ARACNe-AP determines protein-gene regulatory interactions by assessing the statistical significance of mutual information (MI) between the gene expressions of regulator proteins and potential targets. Subsequently, the algorithm eliminates statistically significant candidate targets that violate the data processing inequality. For accurate MI analysis by the ARACNe-AP algorithm, a minimum of 100 expression profiles is typically required. The output of ARACNe-AP consists of the likelihood and the regulatory action mode of each protein-gene interaction. The likelihood is an edge weight, ranging between 0 and 1, corresponding to the scaled MI score that is divided by the maximum MI in all edges. The regulatory action mode is the sign of the association (>0: induction, <0: inhibition) between the protein regulator and its target gene, ranging between −1 and +1, computed by Spearman correlation. For an input of ARACNe -AP, we retrieved publicly available gene expression data of human bronchial and nasal epithelial samples from two studies illuminating diagnostic markers of lung cancer (*37*, *38*) (GEO accession numbers: GSE66499 and GSE80796). The downloaded data had been normalized using robust multiarray analysis (*84*) and scaled into log_2_ intensities. Among the samples, we inferred the networks, only using benign lung disease samples (*n* = 190 for GSE66499, *n* = 196 for GSE80796) in each dataset, separately. Also, we prepared an input of the regulator protein list, consisting of the transcriptional (co)regulaors identified based on the following Gene Ontology (GO) terms: transcription regulator activity (GO:0140110), transcription coregulator activity (GO:0003712), and DNA binding transcription activator activity (GO:0001216). ARACNe-AP was implemented with the setting of 200 bootstraps and Bonferroni-corrected threshold of *P* = 0.05 for detecting statistically significant protein-target interactions. Then, we merged the regulatory networks inferred from two datasets (GSE66499 and GSE80796) by averaging the estimates, namely, likelihoods and regulatory action modes for each protein-gene interaction across the two networks. To ensure accuracy, we limit regulons, a set of target genes for each regulator, to a maximum of 50 genes, as the improvement in the precision of VIPER analyses plateaus beyond this point. In addition, we only considered the edges with a strong weight (i.e., likelihood > 0.25). Consequently, only the 50 most statistically significant targets within each regulon were retained. This cautious approach prevents potential biases in VIPER NES assessment, as NES measured from larger regulons would exhibit higher statistical significance. Similarly, we reverse-engineered gene regulatory networks from scRNA-seq data across four conditions (mock, 1 dpi, 3 dpi, and 6 dpi), using ARACNe-AP. To address the sparsity in single-cell expression, we first created metaCells by merging 10 neighboring cells per condition. ARACNe-AP was then applied to the metaCell expressions, producing a network for each of the four conditions, which were subsequently used in metaVIPER analysis to infer single-cell protein activity profiles, as shown in fig. S3A.

### Generation of host response SARS-CoV-2 signatures

First, we generated differential gene expression signatures by comparing the expression profiles of infected and noninfected cells in each cell type. Cell type–specific clusters were identified by applying cell marker enrichment analysis to Louvain clustering of protein activity profiles, inferred through metaVIPER with scRNA-seq-based networks as previously described. A “bootstrapped approach” was implemented in which we compared *k* randomly selected infected cells at each time point with the noninfected cells nearest to each infected cell, using a two-sample Mann-Whitney *U* test, repeated 100 times. In other words, we generated 100 metaCell pairs, each comparing the mRNA reads of *k* random sampled infected cells and of *k* noninfected cells from each time point, using the Mann Whitney *U* test (Fig. 2A). For the infected-versus-mock signature, the noninfected cells were sampled from the mock condition, based on the distance in the principal components analysis (PCA) space and the single-cell VIPER-inferred protein activity. For the infected-versus-bystander signature, the uninfected bystander cells nearest to the infected cells were sampled from the cells with no viral read detection at the same time point. To determine an optimal *k*, we iterated the above procedure with increasing *k* one-by-one. As *k* increases, the differential gene expression converges, and *k* = 23 was determined as an optimal number. In other words, the optimal value of *k* (*k*_Opt_ = 23) was determined by assessing convergence of the metaCell-based analysis versus analyzing the differential expression of all infected versus all noninfected cells. We integrated the bootstrapped signatures using the Stouffer’s method for each combination of cell type and time point. Differential protein activity was then integrated across all 100 metaCells using Stouffer’s *z*-score integration method. These signatures were then used for further analyses. The approach we implemented consisted of a random sampling of *k* infected cells, which provided the intrinsic advantage of generating signatures that were not affected by the number of viral reads in each infected cell and were thus independent of the specific threshold of number of viral reads (fig. S3A). To better illustrate this point, we generated host-response signatures by progressively increasing the threshold on the number of detected reads and assessed the conservation of the top 50 and bottom 50 MRs presented in the manuscript (aREA enrichment analysis). For this, we used the SIS signature from infected-versus-bystander cells (fig. S3A). As expected, increasing the threshold of viral reads reduced the statistical significance of the conservation of the MRs. However, even when the threshold of viral reads is increased to its maximum, the *P* values of the conservation of the signatures were still highly significant (basal: 10^−23^, ciliated: 10^−38^, secretory: 10^−17^; bars at the right in each of the plots in fig. S3A). This strongly suggested that these signatures are not significantly dependent on a viral read threshold. Similar results are expected if this analysis is performed by comparing signatures of infected-versus-mock control cultures at 3 and 6 dpi.

### Comparative analysis SARS-CoV-2 MR host responses from publicly available scRNA-seq datasets

For the comparison analysis of the host response signatures between the study of Ravindra *et al.* (*34*) and our study, we obtained the dataset deposited in a single-cell data platform called CZ CELLxGENE Discover (*85*) by Ravindra *et al.* (*34*). We applied the same QC filtering metrics and the same protein activity inference approach described above to generate host response signatures. In particular, infected-versus-mock signatures (UIS) were analyzed at 3 dpi, a time point common to both studies, which showed SARS-CoV-2 infection and clear host responses across ciliated, secretory, and basal cells for comparisons. The conservation of MR signatures derived from the study of Ravindra *et al.* (*34*) and our study was assessed through the enrichment analysis of the top 50 and bottom 50 MRs between two signatures using the aREA (*10*) algorithm. During the aREA implementation, the signs for top 50 and bottom 50 MRs were set to +1 and −1, respectively, whereas the weights for individual MRs were set to ones (fig. S2, C and D).

### Hallmark pathway enrichment analysis

To gain insights into the biological pathways activated and deactivated during SARS-CoV-2 infection, we conducted pathway enrichment analysis using hallmark gene sets obtained from Molecular Signatures Database (https://gsea-msigdb.org/) (*39*). NES of hallmark pathways in each host response signature were computed using aREA (*10*) in the VIPER R package. The signs and weights for individual genes for each hallmark set were set to ones during the aREA implementation.

### Enrichment analysis of proviral genes

We compiled a set of proviral host factors from CRISPR-KO assays previously conducted on human lung cancer cell lines (A549 and Calu-3) (*3*, *31*, *32*). Specifically, we identified genes with a *z*-score > 1.5, reported in at least one of three studies [Rebendenne *et al.* (*3*), Daniloski *et al.* (*32*), and Biering *et al.* (*31*)] as proviral genes, where the *z*-score reflects gene essentiality during SARS-CoV-2 infection. Among these host factors, 50 TFs and cofactors were identified. We then conducted enrichment analysis of the 50 TFs and cofactors using the fast GSEA (fGSEA) (*86*) R package, visualizing the results through enrichment plots (the plotEnrichment function). In addition, leading edge proteins were identified as those ranked highest up to the point of maximum enrichment score among the 50 TFs and cofactors.

### Drug perturbation screen of organotypic airway epithelial cultures and PLATE-seq data preprocessing

Drug perturbation assays were performed in fully differentiated organotypic airway epithelial cultures at ALI day 21 and analyzed 24 hours later. *C*_max_ represents the maximum tolerated serum concentration of each drug as determined from established studies. Drug signatures were generated using PLATE-seq, a high-throughput RNA-seq platform that allows simultaneously profiling multiple drugs (*71*). A total of 441 drugs, 20 dimethyl sulfoxide (DMSO), and 20 untreated samples were sequenced over 10 96-well plates with duplicates. Note that drugs in each plate were randomly selected regardless of their MoA. Since samples in 96 wells of each plate were pooled together in PLATE-seq technology, we demultiplexed the reads using the computational tool Sabre (https://github.com/najoshi/sabre/) using the sample barcode identifiers. Then, we applied kallisto (*87*) to quantify read counts and gene expression abundance matrices [e.g., transcripts per million (TPM)] for each plate. In detail, while running kallisto, we first generated the human transcriptome indices from the human genome reference (Homo_sapiens.GRCh38.cdna.all.fa and Homo_sapiens.GRCh38.96.gtf as of 12 March 2019) in Ensembl (*88*), using the kallisto index. The transcriptome indices were used during kallisto quant to quantify pair-end reads of the PLATE-seq data.

### Protein activity inference for PLATE-seq data

We inferred protein activity profiles from the PLATE-seq data, using VIPER (*10*). VIPER computes the normalized, rank-based enrichment score (NES) of the regulon of each protein in genes differentially expressed when comparing treatment versus a control. Statistically significant positive and negative NES values provided by VIPER imply activated or inactivated proteins in the state of interest compared to the control state. Meanwhile, nonsignificant NES scores signify proteins with no significant change in activity. Unlike a conventional GSEA, VIPER uses a probabilistic model to integrate consensus among activated, inhibited, and unclearly regulated targets, considering their differential expression. As a result, using a context-specific network which can enhance interactome fidelity is critical for accurately inferring protein activity. In this work, we used the airway regulatory network, inferred by ARACNe-AP as described above, for identifying protein activity profiles of PLATE-seq data. As part of the VIPER analysis, we computed the differential gene expression profiles resulting from drug treatment compared to the control state (DMSO). To mitigate the impact of technical batch effects on differential gene expression, samples were compared with DMSO conditions within the same plate. The differential expression of gene *i* for sample *j* (DE*_ij_*) was determined using the provided equation

$${\text{DE}}_{\mathrm{ij}}=\frac{g_{\mathrm{ij}}-\text{median}\left(g_{\mathrm{iC}}\right)}{\text{MAD}\left(g_{\mathrm{ik}}\right)}$$

where *g_ij_* is the TPM-normalized expression of gene *i* for a treatment sample *j*, *g_iC_* is the TPM-normalized expression for gene *i* of the DMSO control samples *C*, and MAD stands for median absolute deviation [= median (|*g_ik_* − median(*g_iC_*)| and *k* ∈ *C*]. To minimize denominator noise arising from low expression variance, we replaced instances where MAD(*g_ij_*) < 0.01 with 0.01. Protein activity inference of the PLATE-seq data was computed using the human airway network, in the same manner as done above. For duplicated samples, we averaged the protein activity between them.

### ViroTarget

For the viral-induced host response signature, druggable MRs were identified on the basis of the targets of 1738 known drug inhibitors from DrugBank (*66*). To identify inhibitable MRs in our signature, we excluded those with absent gene expression or negative protein activity. Consequently, the final selection of druggable MRs comprised targets significantly activated (Benjamini-Hochberg corrected *P* value < 0.05) and expressed (read count > 0) in at least one cell type among ciliated, basal, and secretory cells.

### ViroTreat

To predict drugs capable of reversing the host response signature to SARS-CoV-2 infection, we used ViroTreat, a modified version of the OncoTreat pipeline (*18*). The pipeline fundamentally uses two data inputs: (i) a target signature and (ii) drug perturbation signatures. In our study, the target signature comprised the top 25 activated and 25 inactivated MRs derived from the host response scRNA-seq data. Simultaneously, the drug signatures were constructed on the basis of protein activity profiles of all transcriptional regulators using PLATE-seq data. The statistical score of the host response inversion against individual drug signatures was computed using aREA. The NES serves as a measure of similarity, with a more negative value indicating a stronger inversion of the host response signature relative to the corresponding drug signature. This computation used the aREA function, a weighted GSEA algorithm in the VIPER R package. In the aREA implementation, we assigned a value of +1 to the top 25 activated MRs and −1 to the top 25 inactivated MRs in the host response signature. We also applied a penalty to MRs whose activities were consistently activated or deactivated with any drug treatment (i.e., low specificity), reducing their importance by adjusting their weight through a sigmoid function during the aREA application. Last, we predicted drugs through this pipeline, separately to the host response from each cell type: basal, secretory, and ciliated cells.

### Reactome pathway enrichment analysis

To identify pathways significantly modulated by the 11 drugs which were identified as strong host-response inverters by ViroTreat, we computed NES of reactome pathway (*73*) terms in each drug signature. For this, we used the gsePathway function in the ReactomePA (*89*) R package. Before implementation, the drug signature was sorted in descending order of protein activity.
