# Supplementary material for: Identification and targeting of regulators of SARS-CoV-2–host interactions in the airway epithelium
Source: Sci Adv. 2025 May 16;11(20):eadu2079. doi: 10.1126/sciadv.adu2079 (PMC12083520; doi:10.1126/sciadv.adu2079)
Supplement: Supplementary file 1 — Figs. S1 to S7 Tables S1 to S4 [file sciadv.adu2079_sm.pdf]

Science Advances
AAAS

Supplementary Materials for

Identification and targeting of regulators of SARS-CoV-2–host interactions in the airway epithelium

Brooke Dirvin *et al.*

Corresponding author: Andrea Califano, ac2248@cumc.columbia.edu;
Wellington V. Cardoso, wvc2104@cumc.columbia.edu

Sci. Adv. **11**, eadu2079 (2025)
DOI: 10.1126/sciadv.adu2079

This PDF file includes:

Figs. S1 to S7
Tables S1 to S4

a

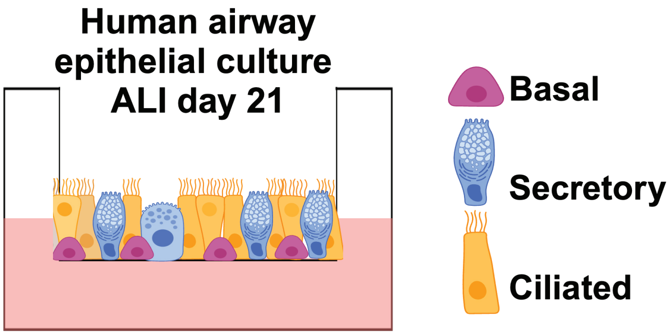

cell-type markers

Basal	Secretory	Ciliated
TP63	MUC5AC	FOXJ1
KRT5	MUC5B	TUBB4B
ITGA6	GP2	TUBA1A
NGFR	SPDEF	CDHR3
PDPN	TFF1	PROM1
KRT17	SCGB1A1	
KRT14	SCGB3A2	
	CYP2F2	

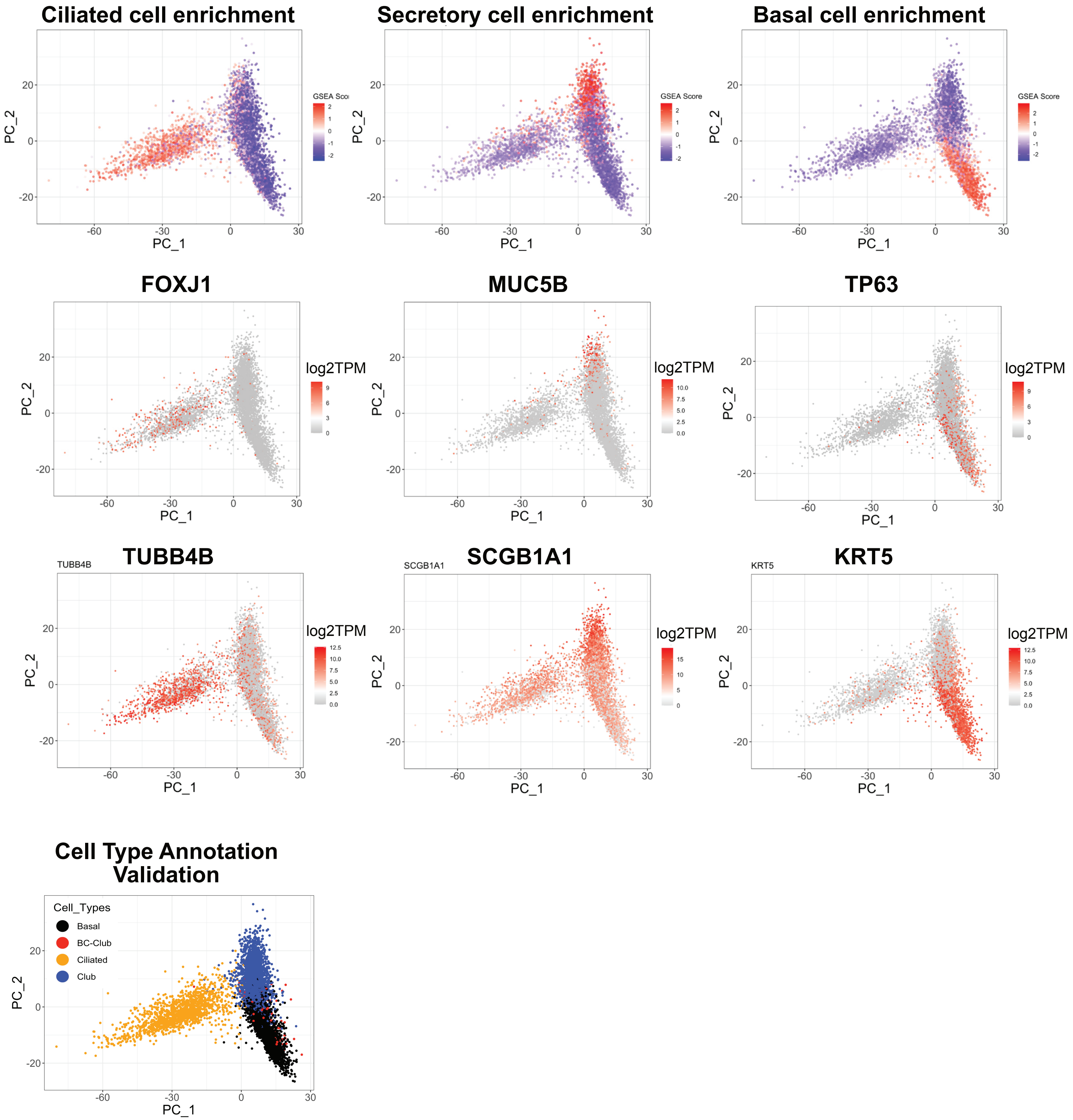

Supplementary Figure 1: Adult human airway epithelial cell populations in control and SARS-CoV-2-exposed ALI cultures

a. Diagram: airway epithelial cell types analyzed in the ALI organotypic cultures and panel of markers used for their identification.
b. Top panels: PCA plots of GSEA results using the gene expression signature in each cell type; high (red) and low (blue) scores are depicted. Lower panels: feature plots of representative marker genes with expression in transcripts per million represented in a gradient from red (high) to gray (low).
c. PCA plot of the independent validation of Cell Type Annotation using an external Single-Cell dataset (Ravindra et al.[34]) as reference to train SingleR[33].

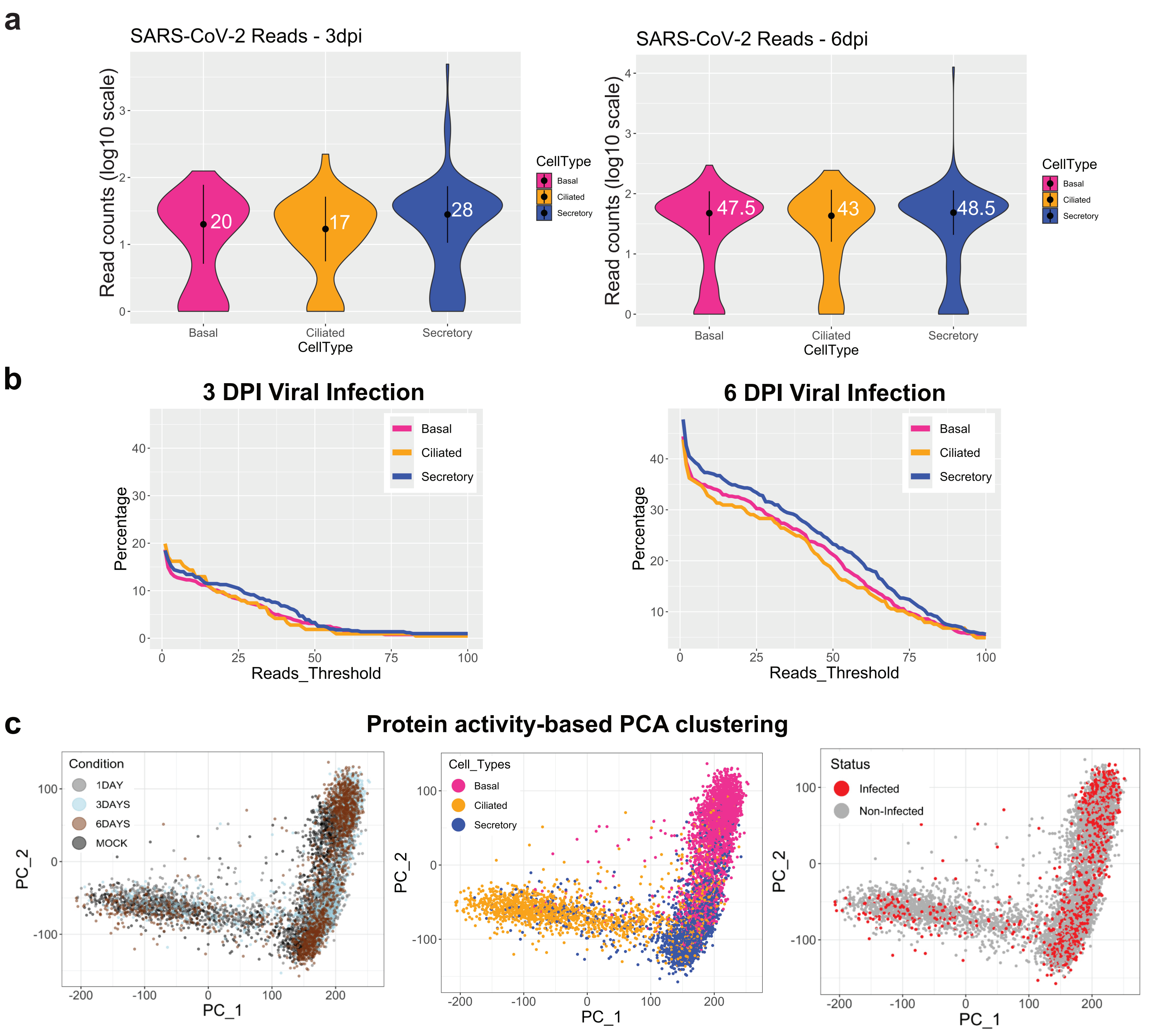

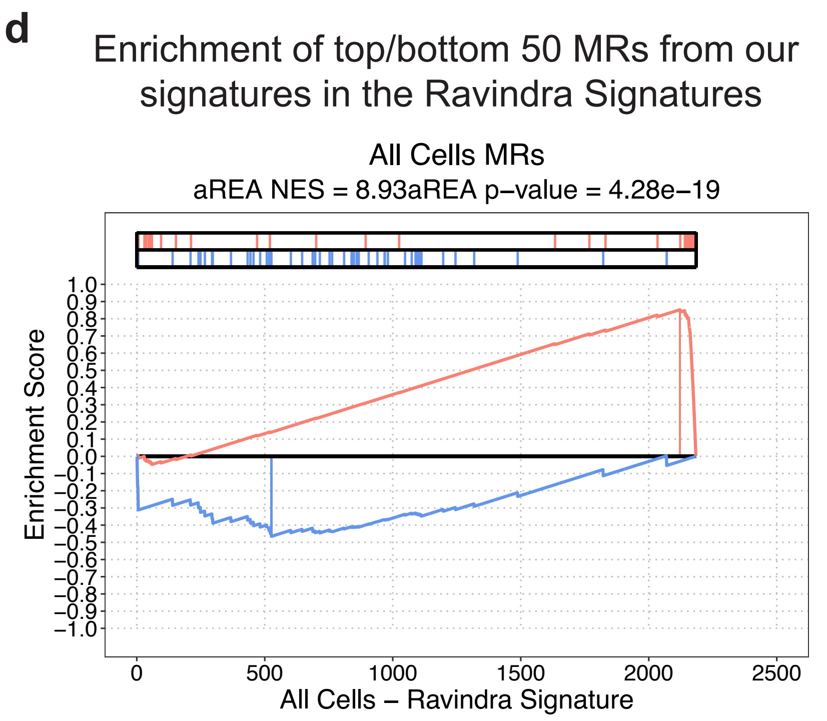

e Cell type-specific enrichment of top/bottom 50 MRs from our signatures in the Ravindra Signatures

	NES	p-value
**Basal**	10.5	6.92e-26
**Ciliated**	13.6	3.73e-42
**Secretory**	3.58	3.48e-4

Supplementary Figure 2: Robustness of the host response MR signature across infected cell thresholds and comparison with Ravindra et al. data.

a. The number of SARS-CoV-2 viral reads detected in infected cells at 3dpi and 6dpi. The read counts are in log10 scale. The values inside the violin plots indicate the median read counts for the SARS-CoV-2 genome.
b. Plot showing the percentage of infected cells (y-axis) in the three different cell types (basal ciliated, secretory) when the threshold of viral reads ranges from 1 to 100 (x-axis) at either 3 or 6 dpi.
c. PCA plots based on protein activity of quality-control filtered cells to demonstrate consistency between protein activity and gene expression. **Left panel:** cells colored according to the timepoints (MOCK, 1 dpi, 3 dpi, 6 dpi). **Middle panel**: cells colored based on cell types: Basal, Ciliated, and Secretory (>75% of the cells showing agreement between gene expression and protein activity. **Bottom panel**: PCA plot based on protein activity data of infected (Red) and non-infected (Grey) cells.
d. A GSEA plot comparing host response MR signatures with the Ravindra et al. data. The aREA algorithm was applied to examine the enrichment of the top 50 and bottom 50 MRs of the averaged UIS signature across all cell types at 3 dpi between our study and the Ravindra et al. data.
e. A table containing the enrichment scores (NES) and their statistical significance (p-value) for the comparison of the top 50 and the bottom 50 MRs from the cell type-specific UIS signatures derived at 3dpi between our study and the Ravindra et al. data. The enrichment score was obtained using the aREA algorithm.

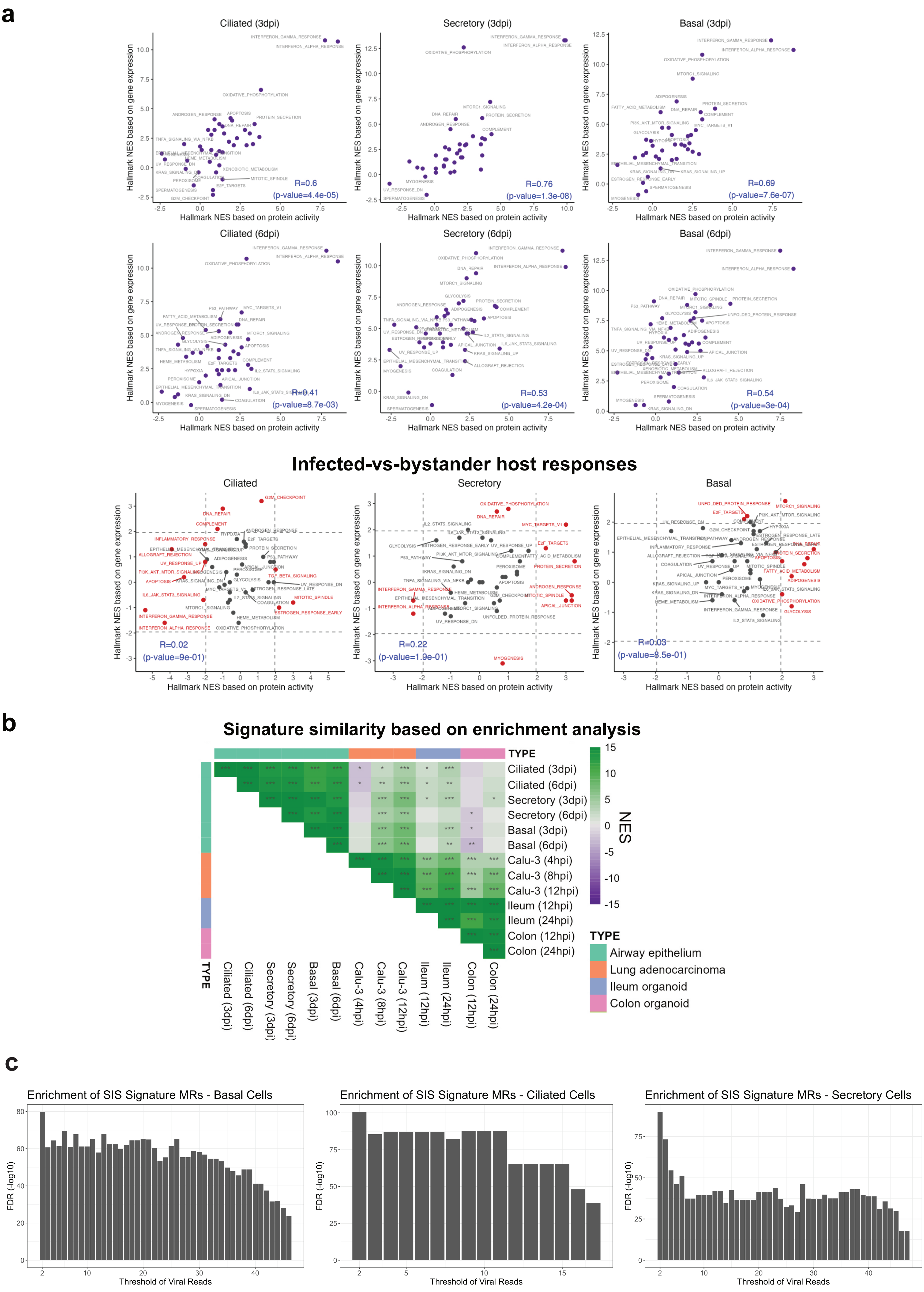
a
Ciliated (3dpi)
Secretory (3dpi)
Basal (3dpi)
R=0.6
(p-value=4.4e-05)
R=0.76
(p-value=1.3e-08)
R=0.69
(p-value=7.6e-07)
Ciliated (6dpi)
Secretory (6dpi)
Basal (6dpi)
R=0.41
(p-value=8.7e-03)
R=0.53
(p-value=4.2e-04)
R=0.54
(p-value=3e-04)
Hallmark NES based on gene expression
Hallmark NES based on protein activity
Infected-vs-bystander host responses
Ciliated
Secretory
Basal
R=0.02
(p-value=9e-01)
R=0.22
(p-value=1.9e-01)
R=0.03
(p-value=8.5e-01)
b
Signature similarity based on enrichment analysis
TYPE
Ciliated (3dpi)
Ciliated (6dpi)
Secretory (3dpi)
Secretory (6dpi)
Basal (3dpi)
Basal (6dpi)
Calu-3 (4hpi)
Calu-3 (8hpi)
Calu-3 (12hpi)
Ileum (12hpi)
Ileum (24hpi)
Colon (12hpi)
Colon (24hpi)
NES
TYPE
Airway epithelium
Lung adenocarcinoma
Ileum organoid
Colon organoid
c
Enrichment of SIS Signature MRs - Basal Cells
Enrichment of SIS Signature MRs - Ciliated Cells
Enrichment of SIS Signature MRs - Secretory Cells
FDR (-log10)
Threshold of Viral Reads

Supplementary Figure 3: Analyses of SARS-CoV-2 host responses in airway organotypic cultures and other cell types.

a. **Top panel**: Scatterplot of hallmark-normalized enrichment score comparisons based on NES protein activity (x-axis) and gene expression analysis (y-axis) in SARS-CoV-2 *treated and mock cells* at 3 and 6dpi. IFN alpha and IFN gamma response pathways were consistently enriched in all cell types by both gene and protein activity. **Bottom panel**: Scatterplot of hallmark-normalized enrichment score comparisons based on NES protein activity (x-axis) and gene expression analysis (y-axis) in SARS-CoV-2 *infected vs uninfected* cells at 3dpi. R and p-values denote a spearman correlation coefficient and its statistical significance, computed using the *stats*[89] package in R.
b. Heatmap showing a comparison of the host response signatures (top 25 activated and top 25 inactivated MRs) of SARS-CoV-2 *infected vs. mock* across different airway epithelial cell types in ALI cultures (3 and 6dpi), human airway-derived lung cancer cells (calu-3) and gastrointestinal organoids (ileum, colon) using aREA analysis (see Methods). Positive (green) and negative (purple) NES values are indicated in heatmap. The asterisk symbols denote the following p-value thresholds: *: $p<0.05$, **: $p<0.01$, and ***: $p<0.001$.
c. Bar plots showing the conservation of the top 50 and bottom 50 MRs of the host response signatures (SIS) generated with an increasing threshold for the number of viral reads for the detection of infected cells using the aREA enrichment analysis (see Methods), The Y -axis denotes the -log10 scaled False Discovery Rate (FDR)-corrected p-values of the enrichment score (NES) between the signature with the threshold=1 and the signature with the corresponding threshold on the x-axis. The SIS signatures were robust over a broad range of viral infection detection thresholds, demonstrating that the statistical significance of MR conservation based on the MR enrichment analysis was better than 10^{-50} even up to the read threshold=20. At the maximum threshold, where the number of detected infected cells decreases dramatically, the MR signature conservation was still highly

significant (see the rightmost bars: Basal: $p=10^{-23}$, Ciliated: $p=10^{-38}$, Secretory: $p=10^{-17}$).

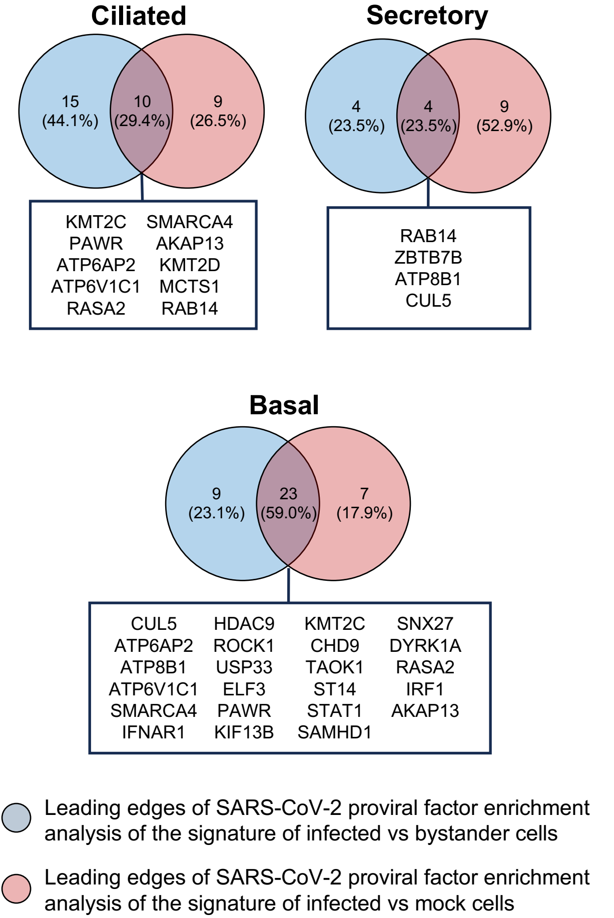
Ciliated
15
(44.1%)
10
(29.4%)
9
(26.5%)
KMT2C
SMARCA4
PAWR
AKAP13
ATP6AP2
KMT2D
ATP6V1C1
MCTS1
RASA2
RAB14
Secretory
4
(23.5%)
4
(23.5%)
9
(52.9%)
RAB14
ZBTB7B
ATP8B1
CUL5
Basal
9
(23.1%)
23
(59.0%)
7
(17.9%)
CUL5
HDAC9
KMT2C
SNX27
ATP6AP2
ROCK1
CHD9
DYRK1A
ATP8B1
USP33
TAOK1
RASA2
ATP6V1C1
ELF3
ST14
IRF1
SMARCA4
PAWR
STAT1
AKAP13
IFNAR1
KIF13B
SAMHD1
Leading edges of SARS-CoV-2 proviral factor enrichment analysis of the signature of infected vs bystander cells
Leading edges of SARS-CoV-2 proviral factor enrichment analysis of the signature of infected vs mock cells

Supplementary Figure 4: Proviral host factors are induced in overlapping and distinct airway epithelial cell types

Venn-diagrams displaying proviral factors enriched in the leading edge of the host response signature of SARS-CoV-2 *infected vs. bystander* (blue) or SARS-CoV-2 *infected vs. mock* (red) in each cell type at 3 dpi. The percentage of proviral factors identified by both approaches (intersection) in each cell type is indicated in ciliated (29.4%), secretory (23.5%) and basal (59.0%) cells.

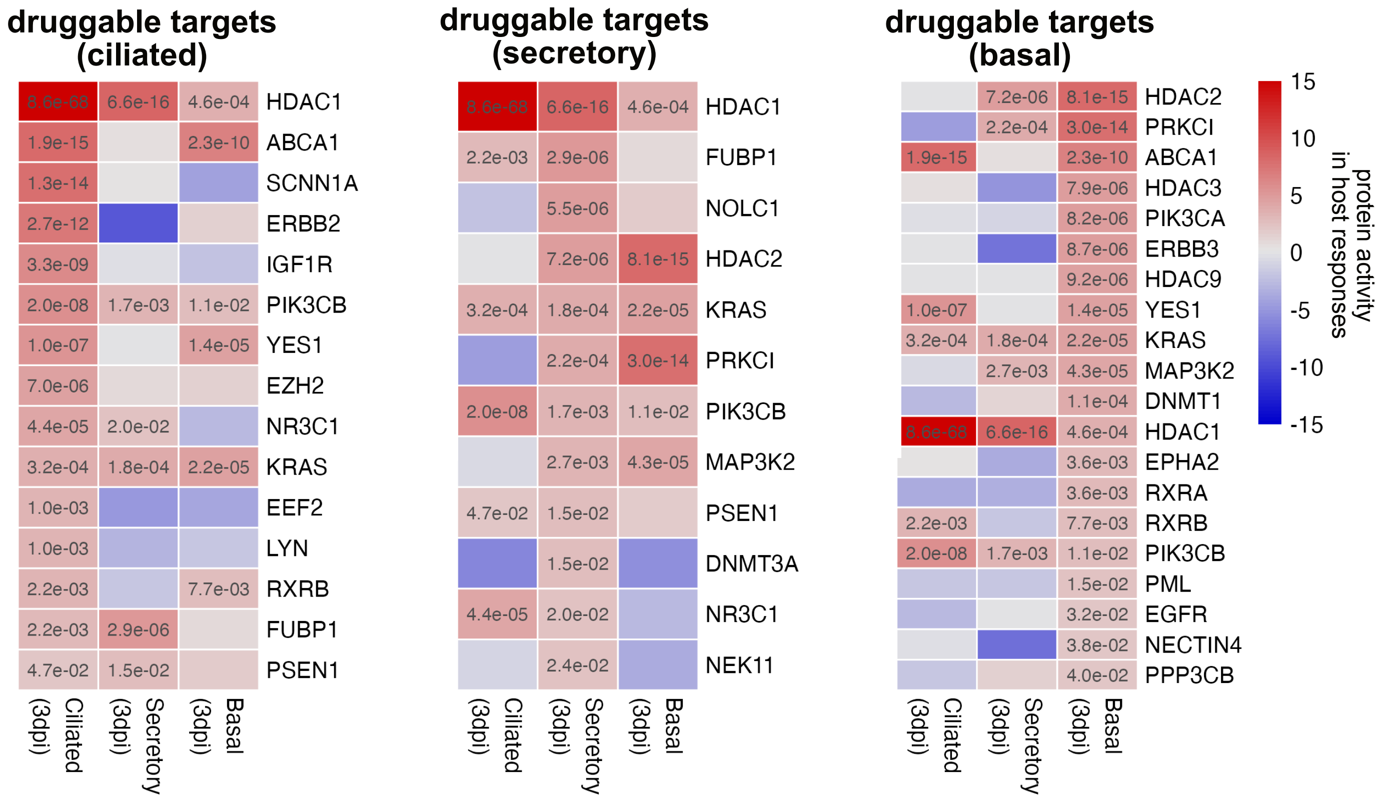

druggable taget	inhibitors
ABCA1	Probucol
DNMT1	Azacitidine, Decitabine, Thioguanine
DNMT3A	Azacitidine, Decitabine
EEF2	Moxetumomab pasudotox
EGFR	Cetuximab, Erlotinib, Panitumumab, Afatinib, Dacomitinib, Osimertinib, Necitumumab, Brigatinib, Mobocertinib, Amivantamab, Gefitinib, Lapatinib, Vandetanib, Neratinib
EPHA2	Dasatinib, Regorafenib
ERBB2	Trastuzumab, Ado-Trastuzumab emtansine, Margetuximab, Tucatinib, Lapatinib, Pertuzumab, Afatinib, Neratinib, Dacomitinib, Osimertinib, Brigatinib
ERBB3	Osimertinib
EZH2	Tazemetostat
FUBP1	Sacituzumab govitecan
HDAC1	Belinostat, Panobinostat, Romidepsin, Vorinostat, Decitabine
HDAC2	Belinostat, Panobinostat, Romidepsin, Vorinostat, Valproic Acid, Decitabine
HDAC3	Belinostat, Panobinostat, Vorinostat, Romidepsin, Decitabine
HDAC9	Valproic Acid, Belinostat, Panobinostat, Romidepsin, Decitabine, Vorinostat
IGF1R	Brigatinib
KRAS	Sotorasib, Adagrasib
LYN	Dasatinib, Bosutinib, Ponatinib, Nintedanib
MAP3K2	Bosutinib
NECTIN4	Enfortumab vedotin
NEK11	Dabrafenib
NOLC1	Doxorubicin
NR3C1	Mifepristone
PIK3CA	Alpelisib, Copanlisib, Idelalisib, Quercetin
PIK3CB	Copanlisib, Idelalisib, Quercetin
PML	Arsenic trioxide
PPP3CB	Pimecrolimus
PRKCI	Tamoxifen
PSEN1	Nirogacestat
RXRA	Bexarotene
RXRB	Bexarotene
SCNN1A	Triamterene, Amiloride
YES1	Dasatinib

Supplementary Figure 5: ViroTarget analysis of druggable MRs of the SARS-CoV-2 host responses.

Heatmap summarizing the druggable MR candidates identified by the ViroTarget algorithm in the SARS-CoV-2 *infected vs. bystander* cells in each cell type (see methods). The heatmap includes druggable MRs enriched in at least one signature of host responses in ciliated, basal, and secretory cells. Their activity is shown in red (activated) and blue (deactivated) across cell types. Candidate drugs identified as an inverter of the activity of each MR are shown on the table below.

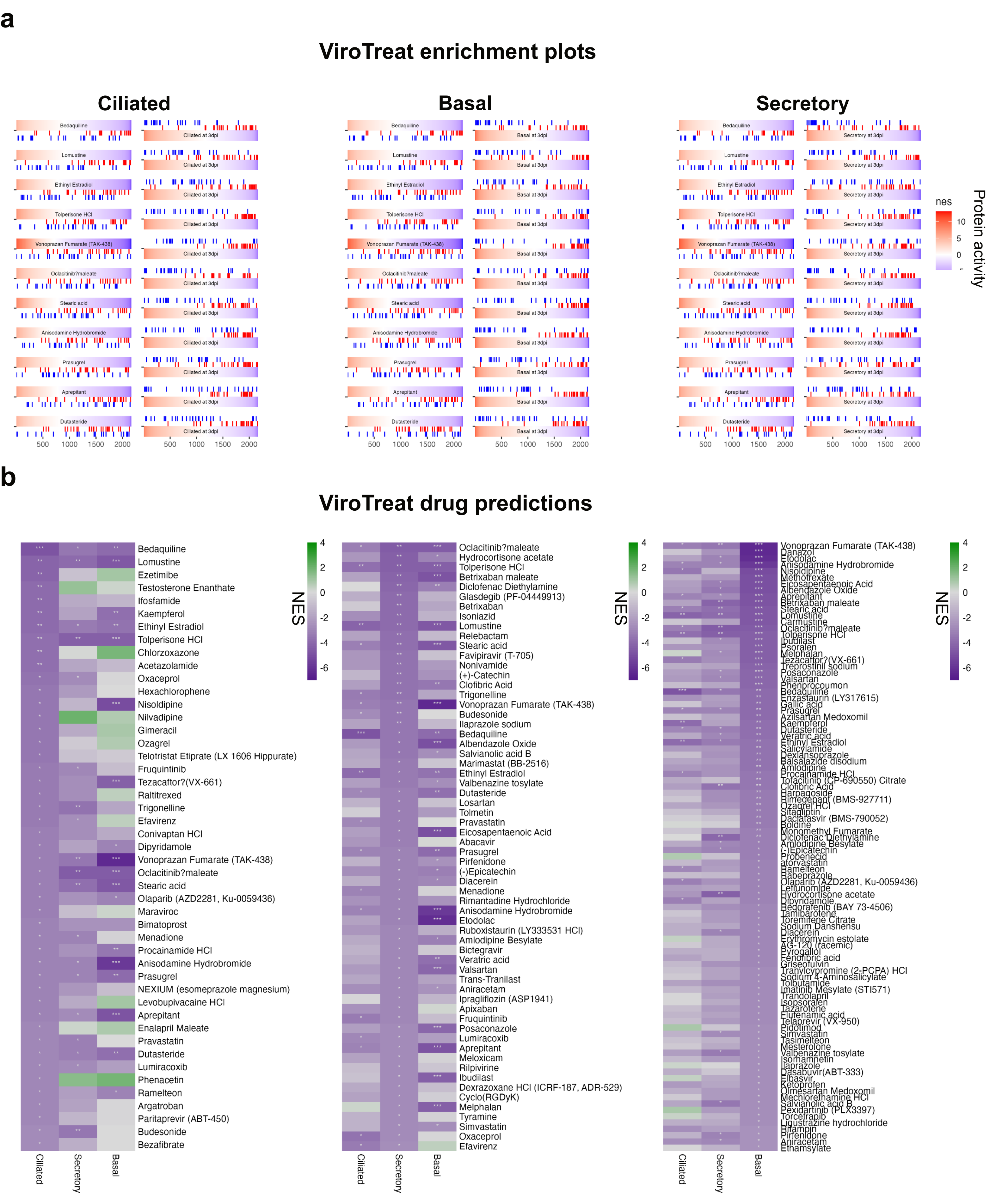

a
ViroTreat enrichment plots
Ciliated
Basal
Secretory
Bedaquiline
Lomustine
Ethinyl Estradiol
Tolperisone HCl
Vonoprazan Fumarate (TAK-438)
Oclacitinib?maleate
Stearic acid
Anisodamine Hydrobromide
Prasugrel
Aprepitant
Dutasteride
Ciliated at 3dpi
Basal at 3dpi
Secretory at 3dpi
500 1000 1500 2000
nes
10
5
0
Protein activity
b
ViroTreat drug predictions
Bedaquiline
Lomustine
Ezetimibe
Testosterone Enanthate
Ifosfamide
Kaempferol
Ethinyl Estradiol
Tolperisone HCl
Chlorzoxazone
Acetazolamide
Oxaceprol
Hexachlorophene
Nisoldipine
Nilvadipine
Gimeracil
Ozagrel
Telotristat Etiprate (LX 1606 Hippurate)
Fruquintinib
Tezacaftor?(VX-661)
Raltitrexed
Trigonelline
Efavirenz
Conivaptan HCl
Dipyridamole
Vonoprazan Fumarate (TAK-438)
Oclacitinib?maleate
Stearic acid
Olaparib (AZD2281, Ku-0059436)
Maraviroc
Bimatoprost
Menadione
Procainamide HCl
Anisodamine Hydrobromide
Prasugrel
NEXIUM (esomeprazole magnesium)
Levobupivacaine HCl
Aprepitant
Enalapril Maleate
Pravastatin
Dutasteride
Lumiracoxib
Phenacetin
Ramelteon
Argatroban
Paritaprevir (ABT-450)
Budesonide
Bezafibrate
Oclacitinib?maleate
Hydrocortisone acetate
Tolperisone HCl
Betrixaban maleate
Diclofenac Diethylamine
Glasdegib (PF-04449913)
Betrixaban
Isoniazid
Lomustine
Relebactam
Stearic acid
Favipiravir (T-705)
Nonivamide
(+)-Catechin
Clofibric Acid
Trigonelline
Vonoprazan Fumarate (TAK-438)
Budesonide
Ilaprazole sodium
Bedaquiline
Albendazole Oxide
Salvianolic acid B
Marimastat (BB-2516)
Ethinyl Estradiol
Valbenazine tosylate
Dutasteride
Losartan
Tolmetin
Pravastatin
Eicosapentaenoic Acid
Abacavir
Prasugrel
Pirfenidone
(-)Epicatechin
Diacerein
Menadione
Rimantadine Hydrochloride
Anisodamine Hydrobromide
Etodolac
Ruboxistaurin (LY333531 HCl)
Amlodipine Besylate
Bictegravir
Veratric acid
Valsartan
Trans-Tranilast
Aniracetam
Ipragliflozin (ASP1941)
Apixaban
Fruquintinib
Posaconazole
Lumiracoxib
Aprepitant
Meloxicam
Rilpivirine
Ibudilast
Dexrazoxane HCl (ICRF-187, ADR-529)
Cyclo(RGDyK)
Melphalan
Tyramine
Simvastatin
Oxaceprol
Efavirenz
Vonoprazan Fumarate (TAK-438)
Danazol
Etodolac
Anisodamine Hydrobromide
Nisoldipine
Methotrexate
Eicosapentaenoic Acid
Albendazole Oxide
Aprepitant
Betrixaban maleate
Stearic acid
Lomustine
Carmustine
Oclacitinib?maleate
Tolperisone HCl
Ibudilast
Psoralen
Melphalan
Tezacaftor?(VX-661)
Treprostinil sodium
Posaconazole
Valsartan
Phenprocoumon
Bedaquiline
Enzastaurin (LY317615)
Gallic acid
Prasugrel
Azilsartan Medoxomil
Kaempferol
Dutasteride
Veratric acid
Ethinyl Estradiol
Salicylamide
Dexlansoprazole
Balsalazide disodium
Amlodipine
Procainamide HCl
Tofacitinib (CP-690550) Citrate
Clofibric Acid
Harpagoside
Rimegepant (BMS-927711)
Ozagrel HCl
Sitagliptin
Daclatasvir (BMS-790052)
Boldine
Monomethyl Fumarate
Diclofenac Diethylamine
Amlodipine Besylate
(-)Epicatechin
Probenecid
atorvastatin
Ramelteon
Rabeprazole
Olaparib (AZD2281, Ku-0059436)
Leflunomide
Hydrocortisone acetate
Dipyridamole
Regorafenib (BAY 73-4506)
Tamibarotene
Toremifene Citrate
Sodium Danshensu
Diacerein
Erythromycin estolate
AG-120 (racemic)
Pyrogallol
Fenofibric acid
Griseofulvin
Tranylcypromine (2-PCPA) HCl
Sodium 4-Aminosalicylate
Tolbutamide
Imatinib Mesylate (STI571)
Trandolapril
Isopsoralen
Tazarotene
Flufenamic acid
Telaprevir (VX-950)
Pidotimod
Simvastatin
Tasimelteon
Mesterolone
Valbenazine tosylate
Isorhamnetin
Ilaprazole
Dasabuvir(ABT-333)
Elbasvir
Ketoprofen
Olmesartan Medoxomil
Mechlorethamine HCl
Salvianolic acid B
Pexidartinib (PLX3397)
Torcetrapib
Ligustrazine hydrochloride
Rifampin
Pirfenidone
Aniracetam
Ethamsylate
Ciliated
Secretory
Basal
NES
4
2
0
-2
-4
-6

Supplementary Figure 6: ViroTreat analysis of druggable MRs of SARS-CoV-2 host responses.

a. ViroTreat enrichment plots for the 11 drugs identified as significant inverters of the host SARS-CoV-2 host response in *infected vs. bystander* (SIS) in each of the cell types indicated
b. Heatmap of candidate drugs predicted by ViroTreat as inverters of the host SARS-CoV-2 host response *in infected vs. bystander* (SIS) cells at 3 dpi in each cell type. Asterisks indicate *$p<0.05$, **$p<0.01$, ***$p<0.001$.

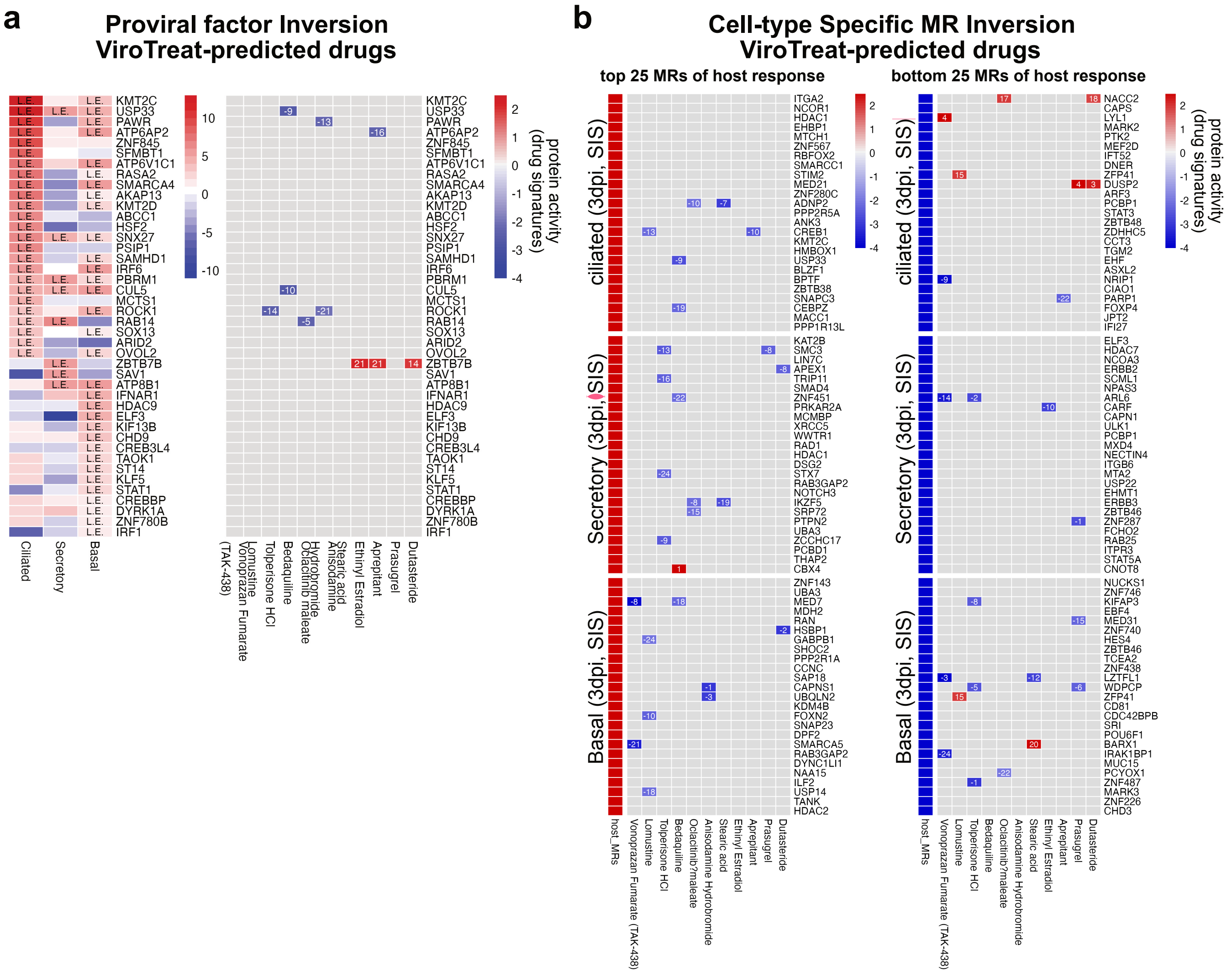

a
Proviral factor Inversion
ViroTreat-predicted drugs
KMT2C
USP33
PAWR
ATP6AP2
ZNF845
SFMBT1
ATP6V1C1
RASA2
SMARCA4
AKAP13
KMT2D
ABCC1
HSF2
SNX27
PSIP1
SAMHD1
IRF6
PBRM1
CUL5
MCTS1
ROCK1
RAB14
SOX13
ARID2
OVOL2
ZBTB7B
SAV1
ATP8B1
IFNAR1
HDAC9
ELF3
KIF13B
CHD9
CREB3L4
TAOK1
ST14
KLF5
STAT1
CREBBP
DYRK1A
ZNF780B
IRF1
Ciliated
Secretory
Basal
L.E.
protein activity
(drug signatures)
Vonoprazan Fumarate (TAK-438)
Lomustine
Tolperisone HCl
Bedaquiline
Oclacitinib maleate
Anisodamine Hydrobromide
Stearic acid
Ethinyl Estradiol
Aprepitant
Prasugrel
Dutasteride
b
Cell-type Specific MR Inversion
ViroTreat-predicted drugs
top 25 MRs of host response
bottom 25 MRs of host response
ciliated (3dpi, SIS)
Secretory (3dpi, SIS)
Basal (3dpi, SIS)
host_MRs
protein activity
(drug signatures)

Supplementary Figure 7: ViroTreat analysis of drugs reversing MRs and proviral factors of the SARS-CoV-2 host responses.

a. Heatmap of the VIPER-inferred top 25 differentially activated (left) and inactivated (right) MR proteins of SARS-CoV-2 *infected vs. bystander* cultures (SIS) at 3 dpi and ViroTreat-inferred top 11 drugs predicted to revert the activity of these MR in each cell type (basal, secretory, ciliated). The MR-reversal scores are represented in the grid if activated (red) or inactivated (blue) by each of the 11 drugs. Numbers in the boxes refer to the rank of the protein in the inverted signature. Reversal of the MRs was statistically significant ($p < 0.05$ Benjamini-Hochberg test).
b. Heatmap of the protein activity of proviral factors found in the leading edge of SARS-CoV-2 *infected vs. bystander* for each cell type at 3dpi (left). MR-reversal of these proviral factors by the top 11 drugs predicted by ViroTreat, represented in the grid as above (right). Reversal of the MRs was statistically significant ($p < 0.05$ Benjamini-Hochberg test).

Supplementary Table 1

Dataset overview: summary of QC statistics

Condition	Quality Control Status	Number of Cells	Mean #UMIs/cell	Mean #Detected Genes/cell
MOCK	Pre-QC	2545	1850.103	824.0998
	**Post-QC**	**1769**	**2293.083**	**974.9638**
1 DPI	Pre-QC	2512	1682.525	774.541
	**Post-QC**	**1628**	**2180.012**	**938.6229**
3 DPI	Pre-QC	1895	2131.499	880.381
	**Post-QC**	**1373**	**2624.546**	**1034.783**
6 DPI	Pre-QC	2396	2947.942	1036.608
	**Post-QC**	**1625**	**3821.113**	**1268.788**
All Conditions	Pre-QC	9348	2143.504	876.6597
	**Post-QC**	**6395**	**2723.742**	**1053.218**

Supplementary Table 2

Cell Populations and infected cells

	MOCK	1 DPI	3 DPI (total)	3 DPI (Infected)	6 DPI (total)	6 DPI (infected)
**Basal**	535	405	643	120 (18.66%)	698	310 (44.41%)
**Ciliated**	503	523	216	43 (19.9%)	265	116 (43.77%)
**Secretory**	731	700	514	95 (18.48%)	662	316 (47.73%)

Supplementary Table 3a

Top Activated and inactivated MRs in the host response signatures **Infected vs Mock**

Top 25 activated proteins

	Ciliated (3dpi)			Ciliated (6dpi)			Secretory (3dpi)			Secretory (6dpi)			Basal (3dpi)			Basal (6dpi)		
rank	protein	NES	FDR	protein	NES	FDR	protein	NES	FDR	protein	NES	FDR	protein	NES	FDR	protein	NES	FDR
1	IFITM1	4.67E+01	0.00E+00	BST2	2.34E+01	1.43E-118	IFITM1	4.87E+01	0.00E+00	MX1	2.29E+01	2.73E-112	IFI6	5.28E+01	0.00E+00	ZNFX1	3.52E+01	1.23E-268
2	IFI6	4.67E+01	0.00E+00	IFI27	2.27E+01	1.00E-111	IFI6	4.76E+01	0.00E+00	ZNFX1	2.20E+01	6.33E-104	MX1	5.00E+01	0.00E+00	IFI27	3.33E+01	1.17E-240
3	IFI27	4.62E+01	0.00E+00	SP110	2.26E+01	4.18E-111	MX1	4.65E+01	0.00E+00	IFI6	2.18E+01	2.42E-102	IFI27	4.88E+01	0.00E+00	IFITM1	3.29E+01	5.56E-235
4	MX1	4.50E+01	0.00E+00	IFITM1	2.09E+01	4.02E-95	ZNFX1	4.63E+01	0.00E+00	SP110	2.11E+01	9.41E-96	ZNFX1	4.82E+01	0.00E+00	SP110	3.20E+01	2.32E-221
5	IRF9	4.48E+01	0.00E+00	ZNFX1	2.09E+01	9.51E-95	IFI27	4.54E+01	0.00E+00	IFITM1	2.10E+01	7.29E-95	SP110	4.60E+01	0.00E+00	IFI6	3.15E+01	1.75E-215
6	ZNFX1	4.31E+01	0.00E+00	IFI6	2.07E+01	8.11E-93	SP110	4.29E+01	0.00E+00	OAS3	2.05E+01	1.57E-90	IFITM1	4.46E+01	0.00E+00	MX1	3.14E+01	3.17E-214
7	PLSCR1	4.02E+01	0.00E+00	IRF9	2.03E+01	4.59E-90	IRF9	4.27E+01	0.00E+00	IFI27	2.04E+01	7.28E-90	STAT2	4.21E+01	0.00E+00	PLSCR1	3.13E+01	1.10E-212
8	SP110	3.96E+01	0.00E+00	PLSCR1	2.03E+01	1.76E-89	OAS3	4.07E+01	0.00E+00	PLSCR1	1.94E+01	4.28E-81	IRF9	4.01E+01	0.00E+00	IRF9	3.06E+01	1.27E-202
9	PARP14	3.91E+01	0.00E+00	CASP7	2.02E+01	6.33E-89	STAT2	3.95E+01	0.00E+00	IRF9	1.93E+01	5.35E-81	OAS3	3.97E+01	0.00E+00	PARP9	2.97E+01	6.94E-192
10	OAS3	3.80E+01	0.00E+00	SP100	2.01E+01	5.54E-88	PLSCR1	3.95E+01	0.00E+00	STAT2	1.92E+01	4.94E-80	PLSCR1	3.78E+01	0.00E+00	PARP14	2.96E+01	1.86E-190
11	STAT2	3.71E+01	5.05E-299	TNFSF10	2.00E+01	5.49E-87	PARP9	3.85E+01	0.00E+00	BST2	1.81E+01	8.25E-71	EIF2AK2	3.74E+01	2.11E-304	SP100	2.91E+01	2.68E-184
12	SP100	3.71E+01	5.31E-299	STAT2	2.00E+01	8.19E-87	PARP14	3.69E+01	1.45E-296	PARP9	1.77E+01	1.18E-67	PARP14	3.69E+01	4.05E-296	OAS3	2.89E+01	4.53E-181
13	TRIM22	3.64E+01	2.70E-287	TRIM22	1.99E+01	3.22E-86	BST2	3.63E+01	7.04E-286	CASP7	1.69E+01	9.67E-62	SP100	3.66E+01	1.37E-291	STAT2	2.88E+01	2.41E-180
14	PARP9	3.60E+01	2.03E-281	KLF13	1.91E+01	2.39E-79	CASP7	3.59E+01	1.12E-279	TRIM22	1.66E+01	9.63E-60	PARP9	3.60E+01	5.02E-281	TRIM22	2.77E+01	1.09E-166
15	BST2	3.55E+01	3.59E-274	PARP14	1.90E+01	3.35E-79	TRIM22	3.51E+01	1.75E-267	SP100	1.65E+01	1.13E-58	TRIM22	3.53E+01	3.88E-270	TNFSF10	2.61E+01	5.20E-148
16	EIF2AK2	3.18E+01	3.13E-219	MX1	1.88E+01	4.81E-77	IFITM3	3.29E+01	1.24E-235	PARP14	1.60E+01	9.69E-56	TNFSF10	3.31E+01	8.92E-239	BST2	2.56E+01	5.94E-142
17	STAT1	3.14E+01	1.93E-214	OAS3	1.82E+01	1.14E-72	SP100	3.24E+01	2.07E-228	IFITM3	1.57E+01	2.77E-53	BST2	3.13E+01	3.42E-213	EIF2AK2	2.54E+01	7.75E-141
18	TNFSF10	3.11E+01	2.21E-210	STAT1	1.79E+01	2.17E-70	ETV7	3.18E+01	2.04E-219	EIF2AK2	1.47E+01	6.37E-47	CASP1	3.13E+01	1.46E-212	CASP1	2.46E+01	1.22E-131
19	CASP1	2.95E+01	1.27E-189	PARP9	1.79E+01	2.27E-70	EIF2AK2	3.13E+01	8.60E-213	TGM2	1.46E+01	3.19E-46	NMI	3.09E+01	1.36E-207	CASP7	2.40E+01	5.34E-125
20	IFITM3	2.93E+01	7.73E-187	FHL2	1.76E+01	4.06E-68	OPTN	3.08E+01	1.03E-206	STAT1	1.45E+01	1.98E-45	IFITM3	2.97E+01	2.90E-191	STAT1	2.37E+01	4.23E-122
21	CASP7	2.75E+01	1.05E-164	TGM2	1.75E+01	3.48E-67	STAT1	3.08E+01	1.30E-206	BCL10	1.43E+01	1.29E-44	OPTN	2.82E+01	2.00E-173	OPTN	2.28E+01	1.58E-113
22	BIRC3	2.63E+01	2.45E-150	CASP1	1.69E+01	6.99E-63	TNFSF10	2.78E+01	1.50E-168	PARK7	1.35E+01	8.16E-40	ETV7	2.81E+01	3.91E-172	ETV7	2.26E+01	1.19E-111
23	ETV7	2.60E+01	1.91E-147	ETV7	1.66E+01	1.22E-60	CASP1	2.50E+01	2.79E-136	HIF1A	1.35E+01	1.31E-39	STAT1	2.72E+01	1.48E-161	TRIM38	2.23E+01	4.98E-108
24	NMI	2.56E+01	1.15E-142	TLE4	1.62E+01	1.72E-57	TGM2	2.28E+01	1.11E-112	CFB	1.34E+01	5.41E-39	TMEM9B	2.61E+01	9.91E-148	NMI	2.22E+01	1.58E-107
25	CCAR1	2.46E+01	1.04E-131	IFITM3	1.61E+01	1.49E-56	BIRC3	2.25E+01	7.60E-110	C3	1.34E+01	7.78E-39	CASP7	2.49E+01	2.32E-135	TMEM9B	2.19E+01	9.84E-105

Top 25 inactivated proteins

	Ciliated (3dpi)			Ciliated (6dpi)			Secretory (3dpi)			Secretory (6dpi)			Basal (3dpi)			Basal (6dpi)		
rank	protein	NES	FDR	protein	NES	FDR	protein	NES	FDR	protein	NES	FDR	protein	NES	FDR	protein	NES	FDR
1	CAPS	-2.26E+01	5.98E-111	SYTL3	-2.47E+01	3.76E-131	RPS6	-3.51E+01	2.44E-268	RPS6	-1.67E+01	2.07E-60	RPS6	-3.23E+01	5.17E-227	UBA52	-2.84E+01	1.75E-175
2	SOX5	-2.24E+01	4.91E-109	SLC22A4	-2.46E+01	4.70E-130	NACA	-2.71E+01	6.65E-160	TOB1	-1.47E+01	5.00E-47	RPS3	-2.96E+01	2.20E-190	RPS6	-2.84E+01	2.92E-175
3	ZNF491	-2.24E+01	7.00E-109	CDHR3	-2.43E+01	1.33E-127	RPS3	-2.48E+01	5.87E-134	DUSP2	-1.42E+01	4.20E-44	NACA	-2.66E+01	3.65E-154	RPS3	-2.55E+01	7.42E-142
4	PROS1	-2.19E+01	1.30E-104	ZNF440	-2.37E+01	7.36E-122	RPL7	-2.27E+01	4.63E-112	NACA	-1.38E+01	1.78E-41	UBA52	-2.56E+01	2.70E-142	NACA	-2.52E+01	9.88E-138
5	ESRRG	-2.15E+01	1.68E-100	IFT172	-2.35E+01	9.38E-120	YBX1	-2.26E+01	4.62E-111	HIPK1	-1.32E+01	4.13E-38	YBX1	-2.33E+01	1.10E-118	POU2AF1	-2.08E+01	1.49E-94
6	RGS22	-2.09E+01	1.18E-95	ZNF491	-2.35E+01	1.84E-119	ZNF599	-1.94E+01	5.97E-82	FOS	-1.32E+01	7.48E-38	RPL7	-2.32E+01	1.10E-117	HES5	-2.03E+01	7.10E-90
7	CERKL	-2.08E+01	8.11E-95	STX2	-2.35E+01	1.86E-119	NPHP1	-1.87E+01	1.30E-76	GADD45B	-1.31E+01	1.48E-37	PRMT1	-1.81E+01	2.75E-71	RPL7	-2.02E+01	5.23E-89
8	SYTL3	-2.06E+01	7.38E-93	HIPK1	-2.34E+01	8.15E-119	TRIM32	-1.84E+01	5.00E-74	RPS3	-1.28E+01	1.82E-35	POU2AF1	-1.72E+01	5.82E-65	YBX1	-1.93E+01	3.49E-81
9	ZNF157	-2.01E+01	1.26E-88	MAK	-2.31E+01	8.12E-116	UBA52	-1.83E+01	2.63E-73	JADE1	-1.27E+01	5.45E-35	MAK	-1.67E+01	4.27E-61	SP5	-1.90E+01	1.54E-78
10	OSBPL6	-2.01E+01	1.91E-88	ZNF19	-2.31E+01	8.12E-116	STOML3	-1.82E+01	6.46E-72	SIX4	-1.26E+01	1.73E-34	PHB2	-1.60E+01	1.56E-56	CATIP	-1.89E+01	6.67E-78
11	ZNF19	-2.01E+01	2.23E-88	TRIP13	-2.30E+01	4.81E-115	LZTFL1	-1.70E+01	4.18E-63	TRIM32	-1.23E+01	2.70E-33	STX2	-1.60E+01	4.77E-56	UNCX	-1.87E+01	2.52E-76
12	IL5RA	-2.01E+01	3.84E-88	NEK11	-2.30E+01	4.81E-115	RFX3	-1.64E+01	1.81E-58	SLC22A4	-1.22E+01	1.85E-32	DLX4	-1.55E+01	5.01E-53	ARHGAP39	-1.86E+01	1.97E-75
13	SLC22A4	-2.00E+01	1.83E-87	DTHD1	-2.28E+01	1.91E-112	LMCD1	-1.64E+01	1.87E-58	TP53BP1	-1.21E+01	5.27E-32	ALX4	-1.54E+01	2.56E-52	ZFHX2	-1.84E+01	3.50E-74
14	STX2	-1.92E+01	6.27E-81	IFT57	-2.26E+01	3.19E-111	THAP10	-1.63E+01	5.69E-58	BCL9	-1.20E+01	9.91E-32	ZBBX	-1.54E+01	3.61E-52	STX2	-1.79E+01	4.78E-70
15	SHANK2	-1.89E+01	2.43E-78	TP53BP1	-2.23E+01	1.94E-108	FOS	-1.63E+01	5.69E-58	MOK	-1.17E+01	9.40E-30	STOML3	-1.52E+01	4.53E-51	UTF1	-1.79E+01	1.21E-69
16	MS4A8	-1.89E+01	8.49E-78	JAZF1	-2.18E+01	9.13E-104	TUSC3	-1.58E+01	1.18E-54	JUNB	-1.16E+01	1.03E-29	ZNF440	-1.44E+01	1.08E-46	LMCD1	-1.78E+01	1.46E-69
17	JADE1	-1.88E+01	1.25E-77	ZNF157	-2.15E+01	8.21E-101	EEF2	-1.58E+01	1.32E-54	UBA52	-1.16E+01	1.61E-29	EEF2	-1.41E+01	4.58E-44	DLX4	-1.78E+01	3.22E-69
18	LMCD1	-1.86E+01	7.16E-76	ZNF3	-2.12E+01	8.80E-98	ZNF440	-1.57E+01	5.01E-54	RORC	-1.15E+01	3.25E-29	ZFP90	-1.41E+01	4.93E-44	STMN1	-1.76E+01	9.95E-68
19	ARHGAP18	-1.77E+01	7.13E-69	MAP3K19	-2.09E+01	6.05E-95	SLC22A4	-1.54E+01	3.19E-52	CERKL	-1.15E+01	6.59E-29	DTHD1	-1.39E+01	1.66E-42	SLC22A4	-1.76E+01	2.15E-67
20	GLIS3	-1.76E+01	7.42E-68	GLIS3	-2.07E+01	2.83E-93	ZNF487	-1.53E+01	1.63E-51	ZFPM1	-1.12E+01	1.98E-27	HES5	-1.38E+01	2.58E-42	ALX4	-1.76E+01	2.15E-67
21	LZTFL1	-1.76E+01	7.85E-68	RP1	-2.06E+01	3.51E-92	DCDC1	-1.52E+01	1.59E-50	DUSP1	-1.11E+01	3.16E-27	SP5	-1.38E+01	3.37E-42	GSC2	-1.75E+01	6.16E-67
22	POU2AF1	-1.74E+01	1.51E-66	ZMYND12	-2.06E+01	5.62E-92	TRIP13	-1.52E+01	2.07E-50	THRA	-1.11E+01	3.90E-27	SLC22A4	-1.38E+01	7.30E-42	SIM2	-1.74E+01	2.83E-66
23	ZNF599	-1.71E+01	4.21E-64	NPHP1	-2.02E+01	5.48E-89	HIPK1	-1.50E+01	2.06E-49	SHANK2	-1.11E+01	4.97E-27	RP1	-1.36E+01	4.00E-41	CDHR3	-1.74E+01	3.27E-66
24	IFT57	-1.65E+01	1.61E-59	DCDC1	-2.00E+01	5.24E-87	TP53BP1	-1.50E+01	2.51E-49	RPL7	-1.10E+01	7.36E-27	RUVBL1	-1.36E+01	5.83E-41	DMRTC2	-1.73E+01	1.05E-65
25	JAZF1	-16.405241	3.72E-59	SIX4	-19.904005	2.22E-86	ZBTB4	-14.802814	4.32E-48	NEK11	-10.874796	4.68E-26	TCEA2	-13.498491	2.32E-40	RAB36	-17.311389	1.20E-65

Supplementary Table 3b

Top Activated and inactivated MRs in the host response signatures **Infected vs Bystander**

Top 25 activated proteins									
	Ciliated (3dpi)			Secretory (3dpi)			Basal (3dpi)		
rank	protein	NES	FDR	protein	NES	FDR	protein	NES	FDR
1	ITGA2	2.07E+01	7.93E-92	KAT2B	1.31E+01	1.24E-35	ZNF143	1.21E+01	3.75E-30
2	NCOR1	1.79E+01	2.63E-68	SMC3	1.28E+01	2.40E-34	UBA3	1.20E+01	4.58E-30
3	HDAC1	1.76E+01	3.12E-66	LIN7C	1.11E+01	5.42E-26	MED7	1.16E+01	2.81E-28
4	EHBP1	1.71E+01	1.32E-62	APEX1	1.01E+01	2.33E-21	MDH2	1.15E+01	8.98E-28
5	MTCH1	1.51E+01	3.43E-49	TRIP11	9.85E+00	2.12E-20	RAN	1.11E+01	6.89E-26
6	ZNF567	1.50E+01	2.02E-48	SMAD4	9.59E+00	2.52E-19	HSBP1	1.04E+01	9.70E-23
7	RBFOX2	1.47E+01	2.20E-46	ZNF451	9.50E+00	5.07E-19	GABPB1	1.02E+01	8.16E-22
8	SMARCC1	1.47E+01	2.47E-46	PRKAR2A	9.36E+00	1.73E-18	SHOC2	1.01E+01	9.69E-22
9	STIM2	1.44E+01	1.60E-44	MCMBP	9.34E+00	1.99E-18	PPP2R1A	9.65E+00	1.01E-19
10	MED21	1.43E+01	8.97E-44	XRCC5	8.58E+00	1.49E-15	CCNC	9.60E+00	1.49E-19
11	ZNF280C	1.37E+01	1.54E-40	WWTR1	8.56E+00	1.58E-15	SAP18	9.44E+00	5.83E-19
12	ADNP2	1.37E+01	2.12E-40	RAD1	8.45E+00	3.67E-15	CAPNS1	9.16E+00	7.05E-18
13	PPP2R5A	1.37E+01	2.42E-40	HDAC1	8.44E+00	3.79E-15	UBQLN2	9.07E+00	1.59E-17
14	ANK3	1.36E+01	5.68E-40	DSG2	8.33E+00	8.21E-15	KDM4B	8.98E+00	3.16E-17
15	CREB1	1.35E+01	1.53E-39	STX7	8.14E+00	3.73E-14	FOXN2	8.96E+00	3.88E-17
16	KMT2C	1.32E+01	6.62E-38	RAB3GAP2	8.13E+00	3.86E-14	SNAP23	8.88E+00	7.41E-17
17	HMBOX1	1.32E+01	9.08E-38	NOTCH3	8.04E+00	7.64E-14	DPF2	8.86E+00	7.85E-17
18	USP33	1.31E+01	2.52E-37	IKZF5	8.01E+00	9.57E-14	SMARCA5	8.86E+00	7.85E-17
19	BLZF1	1.31E+01	4.95E-37	SRP72	8.00E+00	9.85E-14	RAB3GAP2	8.77E+00	1.75E-16
20	BPTF	1.31E+01	4.95E-37	PTPN2	7.91E+00	1.90E-13	DYNC1LI1	8.61E+00	6.75E-16
21	ZBTB38	1.30E+01	1.35E-36	UBA3	7.88E+00	2.38E-13	NAA15	8.54E+00	1.14E-15
22	SNAPC3	1.29E+01	2.73E-36	ZCCHC17	7.86E+00	2.55E-13	ILF2	8.45E+00	2.49E-15
23	CEBPZ	1.26E+01	1.17E-34	PCBD1	7.78E+00	4.95E-13	USP14	8.25E+00	1.28E-14
24	MACC1	1.26E+01	2.12E-34	THAP2	7.67E+00	1.09E-12	TANK	8.19E+00	2.02E-14
25	PPP1R13L	1.26E+01	2.70E-34	CBX4	7.60E+00	1.71E-12	HDAC2	8.14E+00	2.94E-14

Top 25 inactivated proteins									
	Ciliated (3dpi)			Secretory (3dpi)			Basal (3dpi)		
rank	protein	NES	FDR	protein	NES	FDR	protein	NES	FDR
1	NACC2	-1.17E+01	6.68E-30	ELF3	-1.13E+01	6.67E-27	NUCKS1	-1.20E+01	4.58E-30
2	CAPS	-1.14E+01	1.59E-28	HDAC7	-1.11E+01	8.69E-26	ZNF746	-9.66E+00	9.47E-20
3	LYL1	-1.13E+01	5.07E-28	NCOA3	-9.24E+00	4.54E-18	KIFAP3	-9.46E+00	5.32E-19
4	MARK2	-1.12E+01	1.74E-27	ERBB2	-8.95E+00	6.20E-17	EBF4	-9.25E+00	3.28E-18
5	PTK2	-1.12E+01	2.98E-27	SCML1	-8.53E+00	2.01E-15	MED31	-8.25E+00	1.28E-14
6	MEF2D	-1.10E+01	2.00E-26	NPAS3	-8.44E+00	3.71E-15	ZNF740	-7.37E+00	6.90E-12
7	IFT52	-1.09E+01	3.03E-26	ARL6	-8.41E+00	4.41E-15	HES4	-7.33E+00	9.23E-12
8	DNER	-1.08E+01	1.31E-25	CARF	-8.28E+00	1.22E-14	ZBTB46	-7.03E+00	7.09E-11
9	ZFP41	-1.08E+01	1.69E-25	CAPN1	-8.24E+00	1.58E-14	TCEA2	-6.93E+00	1.36E-10
10	DUSP2	-1.07E+01	2.05E-25	ULK1	-7.90E+00	2.09E-13	ZNF438	-6.82E+00	2.71E-10
11	ARF3	-1.04E+01	5.37E-24	PCBP1	-7.70E+00	8.73E-13	LZTFL1	-6.78E+00	3.53E-10
12	PCBP1	-1.04E+01	6.84E-24	MXD4	-7.66E+00	1.09E-12	WDPCP	-6.77E+00	3.69E-10
13	STAT3	-1.02E+01	4.06E-23	NECTIN4	-7.66E+00	1.12E-12	ZFP41	-6.76E+00	3.93E-10
14	ZBTB48	-1.02E+01	4.59E-23	ITGB6	-7.55E+00	2.41E-12	CD81	-6.75E+00	3.97E-10
15	ZDHHC5	-1.01E+01	1.27E-22	MTA2	-7.48E+00	3.44E-12	CDC42BPB	-6.70E+00	5.59E-10
16	CCT3	-1.01E+01	1.46E-22	USP22	-7.48E+00	3.44E-12	SRI	-6.68E+00	6.16E-10
17	TGM2	-1.01E+01	1.59E-22	EHMT1	-7.38E+00	6.73E-12	POU6F1	-6.67E+00	6.63E-10
18	EHF	-9.87E+00	1.27E-21	ERBB3	-7.23E+00	1.84E-11	BARX1	-6.66E+00	6.97E-10
19	ASXL2	-9.83E+00	1.86E-21	ZBTB46	-7.22E+00	1.87E-11	IRAK1BP1	-6.66E+00	6.97E-10
20	NRIP1	-9.79E+00	2.71E-21	ZNF287	-7.12E+00	3.91E-11	MUC15	-6.64E+00	7.84E-10
21	CIAO1	-9.60E+00	1.53E-20	FCHO2	-6.95E+00	1.21E-10	PCYOX1	-6.61E+00	9.52E-10
22	PARP1	-9.58E+00	1.93E-20	RAB25	-6.89E+00	1.83E-10	ZNF487	-6.52E+00	1.68E-09
23	FOXP4	-9.44E+00	6.51E-20	ITPR3	-6.80E+00	3.19E-10	MARK3	-6.40E+00	3.51E-09
24	JPT2	-9.23E+00	4.33E-19	STAT5A	-6.80E+00	3.27E-10	ZNF226	-6.35E+00	4.61E-09
25	IFI27	-9.22E+00	4.96E-19	CNOT8	-6.78E+00	3.54E-10	CHD3	-6.28E+00	7.07E-09

Supplementary Table 4

Top Activated and inactivated MR of drugs predicted by ViroTreat

Top 25 Activated MRs of 11 drugs predicted by ViroTreat

Drug	Bedaquiline		Lomustine		Ethinyl Estradiol		Tolperisone HCl		Vonoprazan Fumarate		Oclacitinib maleate		Stearic acid		Anisodamine Hydrobromide		Prasugrel		Aprepitant		Dutasteride	
Rank	protein	NES	protein	NES	protein	NES	protein	NES	protein	NES	protein	NES	protein	NES	protein	NES	protein	NES	protein	NES	protein	NES
1	CBX4	2.50	PRRX2	2.79	EREG	3.22	AIRE	2.96	HYAL2	4.34	ZNF653	2.90	TRIB3	3.22	MAMSTR	2.48	TRIB3	3.06	EID2	2.53	SMYD1	2.50
2	IRF3	2.48	PQBP1	2.58	GPRC5A	3.19	HNF4A	2.83	AIRE	4.02	MAMSTR	2.66	HES5	3.17	PROP1	2.23	SLC39A4	2.64	ADM	2.52	CDKN1C	2.25
3	RPS3	2.45	LTBR	2.39	FHL2	2.97	DMRTC2	2.82	LMX1A	3.91	HES5	2.45	SP5	3.09	VSX2	2.23	EGR4	2.60	TCF15	2.46	DUSP2	2.25
4	EEF2	2.45	CUX1	2.33	GRHL3	2.92	ONECUT1	2.76	LYL1	3.79	TGFB1I1	2.33	TONSL	3.00	ZNF488	2.15	DUSP2	2.57	SOX12	2.45	DMRTC2	2.13
5	ATP5F1A	2.40	IRF7	2.25	PRRX2	2.73	DMRTB1	2.73	CBFA2T3	3.79	UNCX	2.32	TGFB1I1	2.99	GATA5	2.12	EREG	2.42	ZNF444	2.44	ZNF366	2.13
6	ZNF446	2.37	ELK1	2.17	ZNF446	2.66	POU2F2	2.72	VSX2	3.74	OLIG1	2.28	ZFPM1	2.96	LMX1A	2.07	MAFF	2.41	GSX2	2.33	GSX2	2.06
7	CCNE1	2.29	ZNF219	2.16	IGFBP6	2.63	CBFA2T3	2.71	POU3F2	3.73	FEZF1	2.24	PROP1	2.93	CDKN1C	2.07	ZNF444	2.40	UBA52	2.32	PRRX2	2.05
8	ZMYND10	2.27	PBXIP1	2.16	FEZF1	2.46	KLF13	2.68	HNF4A	3.71	CBFA2T3	2.17	POU3F3	2.86	HOXD13	2.02	KLF1	2.39	DMRTB1	2.30	KCNK1	2.02
9	CTBP2	2.25	NFATC2IP	2.12	AIRE	2.44	VAX2	2.67	CCNE1	3.71	POU3F3	2.16	FOXI2	2.85	CBFA2T3	2.00	ZNF628	2.38	BSX	2.30	TLX2	1.99
10	CITED4	2.17	CRB3	2.08	HR	2.43	TBX10	2.64	SIM2	3.69	ELANE	2.13	UNCX	2.81	DDN	1.99	HIC1	2.35	KLF1	2.28	PRDM12	1.97
11	E4F1	2.14	RAMP1	2.03	CRB3	2.33	FOXG1	2.63	NFATC4	3.69	GATA5	2.12	HMX3	2.77	DTX1	1.96	GRHL3	2.34	EBF1	2.25	TRIB3	1.96
12	PLEC	1.97	HMG20B	2.02	HYAL2	2.31	SLC39A4	2.61	GSC2	3.67	TLE6	2.09	DLX4	2.75	HNF1A	1.94	PAX2	2.34	SLC39A4	2.20	CITED4	1.94
13	PCGF2	1.91	FEZF1	2.02	UNCX	2.27	ZNF488	2.58	BHLHE23	3.63	MAP3K10	2.07	TLX2	2.74	OLIG1	1.88	DMRTC2	2.33	TCF24	2.18	KLF2	1.93
14	HMX3	1.89	CDKN1C	1.99	CFB	2.27	KLF1	2.57	HES5	3.63	SLC22A4	2.06	GSC2	2.74	SMYD1	1.88	VSX2	2.29	SMYD1	2.13	ZBTB7B	1.93
15	RPS6	1.87	ZFP41	1.98	FGFBP1	2.26	PITX2	2.56	DMRTC2	3.59	MYPOP	2.02	BSX	2.72	DRGX	1.88	PIM1	2.22	ZNF488	2.13	MNX1	1.92
16	UBA52	1.87	THRA	1.96	BAX	2.24	TCF23	2.52	TFAP2E	3.56	TBX2	2.00	NR2E1	2.72	ANKRD1	1.88	NR2E1	2.22	NFE2	2.11	FOXE3	1.91
17	ROPN1L	1.86	BAD	1.96	TONSL	2.22	LMX1A	2.50	TBX10	3.53	NACC2	1.99	GATA5	2.70	STMN1	1.87	CD151	2.20	MAZ	2.10	ETV3L	1.88
18	AIRE	1.76	GIPC1	1.95	PAX4	2.22	GSX2	2.48	FGF2	3.53	TONSL	1.96	MAMSTR	2.68	NAMPT	1.86	VAX2	2.18	ELANE	2.10	NACC2	1.87
19	MAZ	1.74	TCF15	1.94	HNF4A	2.20	HOXD9	2.47	TLX2	3.50	SP4	1.96	GFI1	2.68	LHX1	1.84	TCEAL3	2.17	NEUROD2	2.10	ISX	1.84
20	PRDM16	1.74	SLC2A4RG	1.94	LMX1A	2.16	POU3F2	2.46	MAMSTR	3.48	ONECUT1	1.92	BARX1	2.67	PRDM16	1.83	ACTL6B	2.16	SIX5	2.08	IGFBP6	1.82
21	HNF4A	1.74	FOXE3	1.93	ZBTB7B	2.15	VSX2	2.45	STMN1	3.47	EGR4	1.91	PRDM16	2.64	POU2F2	1.83	SOCS3	2.13	ZBTB7B	2.08	LMX1A	1.75
22	PRMT1	1.72	PLEC	1.88	BSX	2.15	FGF2	2.35	ACTL6B	3.42	AIRE	1.90	EGR4	2.62	KLF1	1.81	BSX	2.13	SLC6A8	2.06	NFE2	1.72
23	LMCD1	1.70	SOX12	1.87	MAFB	2.14	STMN1	2.34	ISX	3.41	FHL3	1.89	PURG	2.60	ZFPM1	1.80	SOX18	2.09	AIRE	2.06	DLX2	1.69
24	PRDM13	1.69	DEF8	1.87	TBX10	2.14	PRDM13	2.32	ZFPM1	3.40	ONECUT2	1.88	MED9	2.59	HNF4A	1.77	HYAL2	2.08	FEZF1	2.05	VAX2	1.69
25	TBX2	1.68	MNX1	1.87	ZNF488	2.14	EGR4	2.32	DDN	3.40	PRDM16	1.87	MYPOP	2.58	EGR4	1.77	SOX11	2.08	CBFA2T3	2.05	NEUROD4	1.69

Top 25 Inactivated MRs of 11 drugs predicted by ViroTreat

Drug	Bedaquiline		Lomustine		Ethinyl Estradiol		Tolperisone HCl		Vonoprazan Fumarate		Oclacitinib maleate		Stearic acid		Anisodamine Hydrobromide		Prasugrel		Aprepitant		Dutasteride	
Rank	protein	NES	protein	NES	protein	NES	protein	NES	protein	NES	protein	NES	protein	NES	protein	NES	protein	NES	protein	NES	protein	NES
1	FUBP1	-2.82	RYBP	-3.08	N4BP2L2	-3.54	ZNF487	-3.01	RLIM	-4.32	ZMYM5	-2.58	EVI5	-3.26	CAPNS1	-3.04	ZNF287	-2.60	MTDH	-3.29	GTF2H5	-3.24
2	PRKAA1	-2.81	ITCH	-2.58	DMTF1	-3.15	ARL6	-2.77	IFT88	-4.10	ZNF92	-2.51	NAE1	-3.20	RAB5C	-3.00	ZFP3	-2.59	DNAJA1	-3.02	HSBP1	-3.05
3	MED4	-2.80	DOCK1	-2.54	ZRANB2	-3.09	TBPL1	-2.75	LZTFL1	-4.09	SETD3	-2.41	ZMYM2	-3.10	UBQLN2	-2.90	ZFC3H1	-2.47	CIR1	-2.74	RAB36	-2.97
4	MTA3	-2.73	DLG1	-2.51	TUT4	-3.08	ZNF273	-2.71	PSMA4	-3.98	PIK3C2A	-2.39	ELF2	-3.09	ADRM1	-2.67	SNX4	-2.45	SKIL	-2.73	PSMC3IP	-2.54
5	ZNF322	-2.71	PFDN1	-2.48	SUPT20H	-3.00	WDPCP	-2.56	CDK7	-3.94	RAB14	-2.39	TDRD3	-3.04	TMEM9B	-2.38	NR2C1	-2.43	PSMA4	-2.68	GPR162	-2.53
6	NFXL1	-2.70	OGT	-2.42	ZMYM4	-2.99	CEP290	-2.56	GDI2	-3.83	RLIM	-2.18	CEP89	-3.03	TMEM30B	-2.35	WDPCP	-2.37	RYBP	-2.67	N4BP2L2	-2.40
7	ATRX	-2.67	ELF1	-2.36	GPR162	-2.92	EVI5	-2.54	BIRC2	-3.82	GSTP1	-2.18	ADNP2	-2.98	FUBP1	-2.33	ADNP	-2.35	SERINC3	-2.56	RFX2	-2.39
8	STXBP3	-2.66	SMAD1	-2.34	ZNF292	-2.90	KIFAP3	-2.50	MED7	-3.82	IKZF5	-2.16	METAP2	-2.87	MSRB2	-2.32	SMC3	-2.32	BASP1	-2.56	APEX1	-2.33
9	USP33	-2.66	CHD6	-2.32	DNAAF1	-2.84	ZCCHC17	-2.48	NRIP1	-3.80	ZNF639	-2.15	GTF2A1	-2.86	ARF5	-2.31	PATJ	-2.30	ERBIN	-2.48	DMTF1	-2.26
10	CUL5	-2.65	FOXN2	-2.32	CARF	-2.83	ATRX	-2.47	ZFP3	-3.69	ADNP2	-2.09	SS18	-2.85	NFE2L2	-2.30	ZMYM4	-2.24	CREB1	-2.48	OGT	-2.23
11	CHD1	-2.44	N4BP2L2	-2.27	CDHR3	-2.78	ZNF391	-2.44	IFT57	-3.68	SMAD2	-2.06	CSNK1A1	-2.84	RABL3	-2.29	KLF12	-2.23	PRKDC	-2.45	RPS6	-2.23
12	ZNF189	-2.44	DMTF1	-2.19	SUPT7L	-2.77	DCDC1	-2.43	COPS5	-3.67	GTF2A1	-2.06	LZTFL1	-2.83	NCSTN	-2.29	PRKDC	-2.23	IQGAP1	-2.45	YWHAQ	-2.22
13	MAP4K5	-2.39	CREB1	-2.18	RFX2	-2.77	SMC3	-2.42	SPA17	-3.64	ZNF24	-2.04	SETD3	-2.82	PAWR	-2.29	AEBP2	-2.22	OGT	-2.43	GOPC	-2.21
14	CSNK1G3	-2.38	BRWD1	-2.18	OGT	-2.72	ROCK1	-2.42	ARL6	-3.59	PEX11B	-2.03	URI1	-2.76	DAG1	-2.26	PIAS1	-2.22	EVI5	-2.35	POLR2K	-2.18
15	CD47	-2.36	ZMYM4	-2.16	WWC1	-2.72	ZFC3H1	-2.39	SOD1	-3.58	SRP72	-2.02	BTAF1	-2.76	HDAC3	-2.21	MED31	-2.20	ZSCAN16	-2.35	STYXL1	-2.17
16	LCOR	-2.30	COG3	-2.16	PHIP	-2.68	TRIP11	-2.36	THRAP3	-3.54	UTP11	-2.00	COPS5	-2.72	FOXJ3	-2.16	ZC3H6	-2.18	ATP6AP2	-2.34	PARK7	-2.16
17	NFYB	-2.28	ZNF585A	-2.16	NSD3	-2.63	ZNF599	-2.34	GTF2H5	-3.53	SUMO1	-1.98	CNOT2	-2.71	AP2M1	-2.11	G3BP2	-2.17	IMPA1	-2.34	ZNF491	-2.15
18	MED7	-2.27	USP14	-2.13	RAB36	-2.61	TIAL1	-2.33	ZBBX	-3.50	SMAD5	-1.97	BRD7	-2.68	FOXA1	-2.06	ZNF273	-2.16	UBE2L3	-2.34	CNOT4	-2.13
19	CEBPZ	-2.26	MED14	-2.11	ZNF644	-2.59	CNOT4	-2.32	GTF2A2	-3.50	BCLAF1	-1.97	IKZF5	-2.66	RYBP	-2.04	CREBL2	-2.14	PAFAH1B1	-2.33	SERINC3	-2.11
20	PCGF3	-2.15	ZNF292	-2.11	BASP1	-2.58	KDM3A	-2.30	UBA52	-3.49	SLC31A1	-1.97	STAM	-2.64	NME2	-2.02	ZNF138	-2.13	KDM1A	-2.32	SPA17	-2.10
21	AFF1	-2.13	DNAJA1	-2.11	LCOR	-2.58	ZBBX	-2.30	SMARCA5	-3.48	STRAP	-1.97	PIAS1	-2.64	ROCK1	-2.01	TBPL1	-2.11	ZNF322	-2.32	ZSCAN16	-2.08
22	ZNF451	-2.11	WDR43	-2.09	PIAS1	-2.57	ATR	-2.28	RAB28	-3.46	PCYOX1	-1.96	COPS3	-2.60	MIER1	-2.00	HBP1	-2.11	PARP1	-2.31	PFDN1	-2.06
23	CASP8AP2	-2.09	RABL3	-2.08	SCAI	-2.56	RFX3	-2.26	ZNF267	-3.42	CREBZF	-1.95	PIAS2	-2.60	TLE4	-2.00	TADA1	-2.06	N4BP2L2	-2.31	CDK7	-2.05
24	CGGBP1	-2.08	GABPB1	-2.07	ZFHX2	-2.55	STX7	-2.25	IRAK1BP1	-3.41	KCNK1	-1.94	RICTOR	-2.60	ADIPOR1	-1.99	ZSCAN16	-1.98	TAF11	-2.29	RYBP	-2.05
25	RFC1	-2.07	SUPT3H	-2.05	MLLT10	-2.52	HNRNPK	-2.24	YWHAE	-3.41	COG3	-1.93	RAB18	-2.59	ZNF189	-1.99	ZMYM5	-1.97	BRWD1	-2.28	CDK12	-2.05
